# Genome-wide annotation, comparison and functional genomics of carbohydrate-active enzymes in legumes infecting *Fusarium oxysporum formae speciales*

**DOI:** 10.1080/21501203.2019.1706656

**Published:** 2020-01-04

**Authors:** Abhijeet Roy, Aiswarya Jayaprakash, Raja Rajeswary T, A. Annamalai, PTV Lakshmi

**Affiliations:** a Centre for Bioinformatics, School of Life Sciences, Pondicherry University, Puducherry, India; b PG and Research Department of Botany, Arignar Anna Government Arts College, Villupuram, India

**Keywords:** Legumes, Fusarium wilt, *formae speciales*, CAZymes, PCWDE, transcriptomics

## Abstract

Fusarium wilt caused by soil borne ascomycetes fungi *Fusarium oxysporum *which has host-specific forms known as *formae speciales* (ff. spp.), apparently requires plant cell wall degrading enzymes (PCWDE) for successful invasion. In this study, 12 *F. oxysporum* ff. spp. were taken for genome-wide annotation and comparative analysis of CAZymes, with an assessment of secretory PCWDE and orthologues identification in the three legumes infecting ff. spp.  Further, transcriptomic analysis in two legumes infecting ff. spp. using publically available data was also done. The comparative studies showed Glycoside hydrolase (GH) families to be abundant and Principle Component Analysis (PCA) formed two distinct clusters of ff. spp. based on the CAZymes modules and families. Nearly half of the CAZymes in the legumes infecting ff. spp. coded for signal peptides. The orthologue clusters of secretory CAZymes common in all the three legume infecting ff. spp. mostly belonged to families of AA9, GH28, CE5 and PL1 and the expression analysis revealed the abundant PCWDE were differentially expressed in these legumes infecting ff. spp.  Therefore, this study gave an insight into the distribution of CAZymes especially extracellular PCWDE in legumes infecting ff. spp. with further shedding light onto some of the key PCWDE families through differential expression analysis.

## Introduction

Most of the fungal pathogens of ascomycetes are necrotrophic including *Fusarium oxysporum*, a soil-borne wilt causing pathogen which affects the host plant mainly through root by colonising the treachery elements and subsequently results in wilting of aerial part, yellowing of leaves, necrosis and death (Leslie and Summerell 2006; Nene et al. 2012). This fungal pathogen exists in host-specific forms known as *formae speciales* (ff. spp.) singular *formae specialis* (f. sp.) which are also believed to have evolved into many races and within these *formae speciales, F. oxysporum* f. sp. *lycopersici* with its host, i.e. tomato, is considered as model system for molecular studies of resistance and susceptibility of plant (Takken and Rep 2010; Essarioui et al. 2016). Leguminous plants including chickpea, pea and alfalfa are of high nutritional and economic values where the former two are considered as second and third largest important legume crops worldwide (FAO:www.fao.org) and are prone to be affected by f. sp. *ciceris* (Haware et al. 1986), f. sp. *pisi* (Hepple 1963) and f. sp. *medicaginis* (Rhodes 2015) respectively. These *formae speciales* exhibit different races on the basis of their virulence or differential reaction to host cultivars (Correll 1991); for instance f. sp. *pisi* has four races (race 1,2,5 and 6) where race 1 and 2 have been reported frequently in almost every country (Infantino et al. 2006) and for f. sp. *ciceris*, eight races have been reported worldwide, i.e. race 0, 1A, 1B/C, 2, 3, 4, 5 and 6 (Del Mar Jiménez-Gasco et al. 2001), where race 1 is highly pathogenic in India (Singh et al. 2010) and devastating, if unchecked. Although no races have been observed for the f. sp. *medicaginis*, still it is used as a model legume pathosystem (Williams et al. 2016) due to *Medicago* being model species in the fabaceae family.

All the phytopathogens synthesise Plant Cell Wall Degrading Enzymes (PCWDE) (Blackman et al. 2014; Kubicek et al. 2014) and are identified as the Carbohydrate-active Enzymes (CAZymes) (Lombard et al. 2014). The Carbohydrate-active Enzymes (CAZymes) present as different modules deposited in CAZy database (www.cazy.org) are the classes of enzymes required for the synthesis, modification and degradation of polysaccharides and are further classified as glycoside hydrolase (GH) (Henrissat 1991; Henrissat and Bairoch 1993; Henrissat and Davies 1997), glycosyltransferase (GT) (Campbell et al. 1997; Coutinho et al. 2003), Carbohydrate esterase (CE) (Lombard et al. 2010), polysaccharide lyase (PL) (Garron and Cygler 2010; Lombard et al. 2010), auxillary activities (AA) (Levasseur et al. 2013) and Carbohydrate-binding modules (CBM) (Boraston et al. 2004). Currently, glycoside hydrolase (GH) consisting of 161 families, plays a role in hydrolysing glycosidic bonds between two or more carbohydrates or between a carbohydrate and a non-carbohydrate component while glycosyltransferase (GT) with 106 families, helps in the formation of glycosidic bond and biosynthesis of di-, oligo- and polysaccharides whereas Carbohydrate esterase (CE) containing 16 families, catalyzes and splits the ester into respective acid and alcohol. Polysaccharide lyase (PL) consisting of 36 enzyme families cleaves the polysaccharide containing uronic acid via β-elimination mechanism. Meanwhile, enzymes identified to show Auxiliary activities (AA) are distributed to16 families and are involved in redox reaction in conjunction with other CAZymes, where 9 families of it are involved in lignolytic activity and 7 families in lytic polysaccharide mono-oxygenase (LPMO) activities. Some of the CAZymes function as Carbohydrate-Binding Modules (CBM) which have a carbohydrate-binding activity and are reported to constitute approximately 84 families. Thus, these CAZymes modules are required for both growth (Caracuel et al. 2005) and pathogenesis (Jorge et al. 2006; Gibson et al. 2011) while the families of GH, CE, PL and AA involved in cellulose, hemicelluloses, pectin and lignin degradation are reported to be crucial for invasion and successful pathogenesis (Kubicek et al. 2014; Berlemont 2017; Sista Kameshwar and Qin 2017). Besides these, some families of AA also assist GH for degradation of polysaccharides and are known as Lytic Polysaccharide Mono-oxygenase (LPMO) which are also crucial in plant cell wall degradation (Agger et al. 2014; Beeson et al. 2015; Courtade et al. 2016).

Therefore, the PCWDE produced by ascomycetes phytopathogens (Glass et al. 2013; Rytioja et al. 2014) are revealed to be secretory in nature and involved in pathogenicity to establish their own survival (Gibson et al. 2011; Zhao et al. 2013; Kubicek et al. 2014). Perhaps, these PCWDE help in depolymerisation of plant cell wall components composed of cellulose, hemicellulose and pectin (Kubicek et al. 2014) when the pathogens penetrate and invade and also to sustain in the host tissue (Gibson et al. 2011).

Though the genomic sequence for the above-mentioned legumes infecting ff. spp. have been accomplished by Williams et al. (2016) who further revealed GH3 and GH43 alone to be representative of dispensable scaffolds in these legumes infecting ff. spp. and in the meanwhile, Joint Genome Institute (JGI) Mycocosm database (genome.jgi.doe.gov/mycocosm/home) (Grigoriev et al. 2014) provides CAZyomes information only on *F. oxysporum* f. sp. *lycopersici* and f. sp. *pisi* till date. This necessitates the exploration of these CAZymes based on genome-wide distribution of plant cell wall degrading enzymes in different *F.oxysporum* ff. spp. Thus, the present study was focused on 12 *F. oxysporum* ff. spp. subjected to annotation and comparative study of CAZymes distributed among their genome with further exploration of PCWDE and secretory CAZymes in the three legumes infecting ff. spp. (f. sp. *ciceris*, f. sp. *medicaginis* and f. sp. *pisi*). However, comparative expressional studies using the publically available RNA-seq data based on different nutritional medium for legumes infecting ff. spp with respect to the f. sp. *lycopersici* were performed to assess the expressional changes of CAZymes. Accordingly, extracellular secretory enzymes having a role in plant cell wall degradation were assessed to shed the light on the distribution and identification of common orthologues among the legumes infecting ff. spp. as well the key CAZymes which showed significant differential expression in two of the legumes infecting *formae speciales*.

## Materials and methods

### Data retrieval

Genomic assemblies of two legumes infecting *Fusarium oxysporum* f. spp.: *F. oxysporum* f. sp. *ciceris* (PRJNA282695) and *F. oxysporum* f. sp. *medicaginis* (PRJNA294248), with protein sequences of 10 *Fusarium oxysporum* ff. spp. including *F. oxysporum* f. sp. *lycopersici* (PRJNA18813), *F.oxysporum* f. sp. *radicis-cucmerinum* (PRJNA306247), *F. oxysporum* f. sp. *cepae* (PRJNA338256), *F. oxysporum* f. sp. *pisi* (PRJNA72771*), F. oxysporum* f. sp. *conglutinans* (PRJNA73543), *F. oxysporum* f. sp. *cubense* (PRJNA174274), *F. oxysporum* f. sp. *radicis-lycopersici* (PRJNA73535), *F. oxysporum* f. sp. *vasinfectum* (PRJNA73537), *F. oxysporum* f. sp. *melonis* (PRJNA73541), *F. oxysporum* f. sp. *raphani* (PRJNA73545) were downloaded from NCBI database (www.ncbi.nlm.nih.gov).

To understand the effect of nutrition on CAZymes expression, RNA-seq data for *F. oxysporum* f. sp. *lycopersici* grown in minimal medium at 28ºC with three replicates (PRJNA450835), *F. oxysporum* f. sp. *medicaginis* grown in potato dextrose broth (PDB) at 22ºC with three replicates (PRJNA294248) and *F. oxysporum* f. sp. *pisi* grown in both rich and minimal medium pooled with one replicate (PRJNA538191) were taken from EBI ENA database (https://www.ebi.ac.uk/ena).

### Gene prediction, CAZymes annotation and data analysis

Since the gene and protein information for f. sp. *ciceris* and f. sp. *medicaginis* were not available on NCBI database, genes and their corresponding proteins were predicted using webAUGUSTUS (Hoff and Stanke 2013) gene prediction server (http://bioinf.uni-greifswald.de/webaugustus/) which has a web-based interface by keeping *Fusarium oxysporum* f. sp. *lycopersici* reference sequences data as a training dataset.

The protein sequences from 12 ff. spp. were used for CAZYmes annotation on dbCAN2 (Zhang et al. 2018) (http://cys.bios.niu.edu/dbCAN2/) which has three integrated automated annotation tools namely – HMMER (Finn et al. 2011) to search against the dbCAN HMM (hidden Markov model) database, DIAMOND (Buchfink et al. 2015) to search against CAZy pre-annotated CAZymes sequence database and HotPep (Busk et al. 2017) to search against the conserved CAZymes short peptide database with their default parameters and suggests that CAZymes annotated from at least two or more tools should be considered for higher confidence in the prediction. Thereafter, hierarchical clustering of the CAZymes families copy numbers was done for these 12 ff. spp. using Cluster3 (de Hoon et al. 2004) and visualised through Java Treeview software. Principal component analysis of these ff. spp. based on CAZymes modules and the copy numbers of each CAZymes families were also done using an online tool ClustVis (Metsalu and Vilo 2015) (https://biit.cs.ut.ee/clustvis/) with unit variance scaling applied to the row and singular value decomposition (SVD) imputation for principal components (PC). PC1 and PC2 were considered for explained variation and represented as 2D plot. Also, the copy number variations in PCWDE were manually assessed for three legume infecting *formae speciales*.

### Identification of secretory CAZymes

All the predicted CAZymes in *F. oxysporum* f. sp. *ciceris* affecting chickpea and other two legumes infecting ff. spp.- f. sp. *medicaginis* and f. sp. *pisi*. were searched for signal peptides using signalP 4.1 (Nielsen 2017) (http://www.cbs.dtu.dk/services/SignalP-4.1/) with default parameters using D-cut off value of 0.45 and 0.50 for signal peptide with no transmembrane (signalP-noTM) and signal peptide with transmembrane (signal-TM) respectively. Also, the sub-cellular localisations were predicted using DeepLoc1.0 (Almagro Armenteros et al. 2017) (http://www.cbs.dtu.dk/services/DeepLoc/) by keeping search option to PROFILES for accurate prediction and thereafter sorted manually for extracellular soluble CAZymes.

### Orthologues identification

Orthologous studies of the protein sequences identified as extracellular soluble CAZymes in three of the legumes infecting ff. spp. were done using Orthovenn (Wang et al. 2015) (http://www.bioinfogenome.net/OrthoVenn/), which is a web-based tool for annotation and prediction of orthologues in the organisms by keeping the default parameters. The results were generated as cluster Venn diagram with list of orthologues and their GO annotations.

### Differential expression analysis

To find out the expressional changes in CAZYmes among legumes infecting ff. spp. and their comparison with the abundant CAZYmes annotated in genomic assemblies, three publically available RNA-seq data (as mentioned in data retrieval section) were taken and subjected to differential gene expression analysis by comparing two legumes infecting ff. spp against f. sp. *lycopersici* using Galaxy suite (https://usegalaxy.org/) (Giardine et al. 2005), where initial FastQC (http://www.bioinformatics.babraham.ac.uk/projects/fastqc/) was done followed by HISAT2 (Kim et al. 2015) for reads alignment, FeatureCount (Liao et al. 2014) for reads quantification and DESEQ2 (Love et al. 2014) for differential expression analysis. The differentially expressed genes (DEGs) were filtered using FDR cut-off <0.05 and logFC ≥ |2| and after comparison of DEGs in f. sp. *medicaginis* vs. f. sp. *lycopersici* and f. sp. *pisi* vs. f. sp. *lycopersici*, the common accessions were annotated for CAZymes and identified for the presence of secretory CAZymes within these two legumes infecting ff. spp.

## Results and discussion

### Gene prediction

Genome and protein sequences of all the 12 *F. oxysporum* ff. spp. were retrieved from NCBI database and summed up in details containing strain, family, genome size, host and total protein content (Supplementary Table S1). In this study, a total of 16,414 genes were predicted in the f. sp. *ciceris* genomic assembly while 15,741 genes were predicted in f. sp. *medicaginis* genomic assembly and all these predicted sequence information can be accessed through figshare (10.6084/m9.figshare.8124359). Protein sequences of the predicted genes from f. sp. *ciceris* and f. sp. *medicaginis* along with other 10 ff. spp. were taken further for CAZymes annotation.

### CAZymes annotation and distribution

The overall distribution of CAZymes in each of the ff. spp. generally coded for approximately 3% of the total protein-coding sequences as already evidenced by Davies et al. (2005), according to whom in most of the prokaryotes and eukaryotes, the percentage of CAZymes ranged between 1% and 3% of the total protein-coding genes.

**Figure 1.**
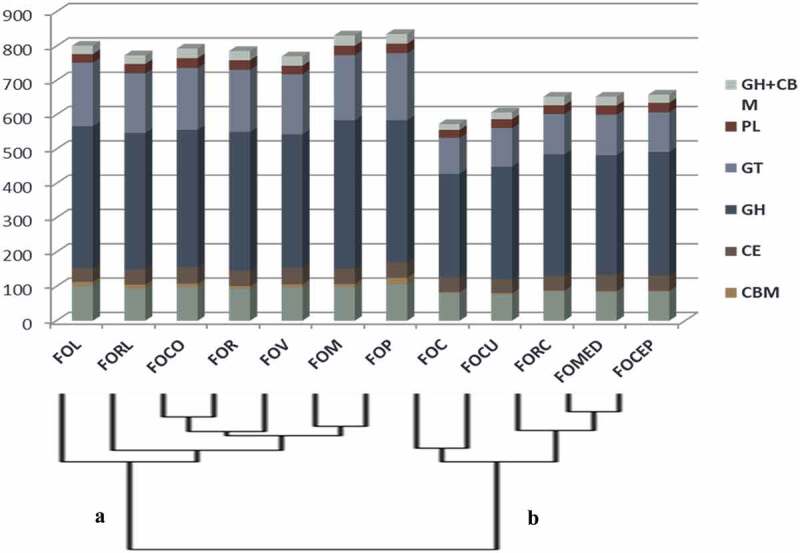
Relative frequency distribution of CAZymes modules and their families in 12 *F. oxysoprum formae speciales.* (Species represented in the x-axis, clade A includes: FOL: *F. oxysporum* f. sp. *lycopersici*, FORL*: F.oxysporum* f. sp. *radicis-lycopersici*, FOCO: *F. oxysporum* f. sp. *conglutinans*, FOR: *F. oxysporum* f. sp. *raphani*, FOV: *F. oxysporum* f. sp. *vasinfectum*, FOM: *F. oxysporum* f. sp. *melonis*, FOP: *F. oxysporum* f. sp. *pisi* and clade B includes: FOC: *F. oxysporum f*. sp. *ciceris*, FOCU: *F. oxysporum* f. sp. *cubense*, FORC: *F. oxysporum* f. sp. *radicis-cucmerinum*, FOMED: *F. oxysporum* f. sp. *medicaginis*, FOCEP*: F. oxysporum* f. sp. *cepae*)

The sequences of CAZymes distributed among all the 12 ff. spp., revealed the highest of 835 and lowest of 573 CAZymes in f. sp. *pisi* and f. sp. *ciceris* respectively by considering at least two of the prediction tools of dbCAN2. CAZymes module CBM was feebly represented and encoded for just 0.5–2.2% in all the ff. spp., while GH was leading to the highest (52%) of the total CAZymes followed by GT, AA, CE, PL and CBM (Figure 1). The copy numbers of GH were found to be highest (432) in f. sp. *melonis* and lowest (301) in f. sp. *ciceris*. Likewise, comparison of the CAZymes distribution in all ff. spp. also showed f. sp. *pisi* to outperform the others. For instances, the highest GT was observed in f. sp. *pisi* (195) while the lowest was in f. sp. *ciceris* (105). Similarly, modules AA (106) and CBM (19) were also highest in f. sp. *pisi* and the other modules such as PL (29) and CE (49) were observed to be higher in *f. sp. conglutinans* and f. sp. *vasinfectum* respectively (Supplementary Table S2).

The families within the modules were sorted and accordingly, module GH represented 79 different families where 12 of them were associated with CBM, while GT, AA, CE, PL and CBM each represented 31, 13, 9, 6 and 3 families, respectively. Within the GH module among all the ff. spp., f. sp. *pisi* (49), f. sp. *raphani* (40) and f. sp. *melonis* (34) over-represented the CAZymes families with their copy number ranging between 27 and 49 for GH3, 23 and 40 for GH16 and 24 and 34 for GH18, respectively. In the meanwhile, GH5 exhibited an average copy number of 22 in all the ff. spp. GH13 was higher in f. sp. *melonis* (18), GH32 in f. sp. *lycopersici* (13) and GH31 with an average copy number of 9 in all the ff. spp. Out of the total CAZymes coding CBM along with GH families, only GH78 associated with CBM67 exhibited higher copy number ranging from 8 to 14 among all the ff. spp.

In GT module, GT2 and GT1 over-represented with copy numbers ranging from 22 to 48 and 12 to 22 and high in f. sp. *pisi* (48) and f. sp. *melonis* (22), respectively, whereas GT4 and GT8 that ranged between 6–19 and 7–14 was revealed to be high in f. sp. *conglutinans* (19) and f. sp. *lycopersici* (14) respectively. In module AA, the family AA3 was ranging highest (25–36) followed by AA1 (14–21) and AA9 (13–20) each represented high copy number within f. sp. *pisi* (36), f. sp. *radicis-lycopersici* (21) and f. sp. *vasinfectum* (20), respectively. In CE module, CE4 and CE5 were found to be higher in copy number with an average of 9 in all these ff. spp. In PL module, PL1 with copy number ranging from 10 to 14 was highest with f. sp. *cepae* (14) having the highest copy number followed by PL3 with an average copy number of 7 in all ff. spp. Unlike the others, CBM21 of CBM module exhibited the highest copy number of 8 in two of the ff. spp. including f. sp. *conglutinans* and f. sp. *pisi* (Supplementary Table S3).

### Principle Component Analysis (PCA)

**Figure 2.**
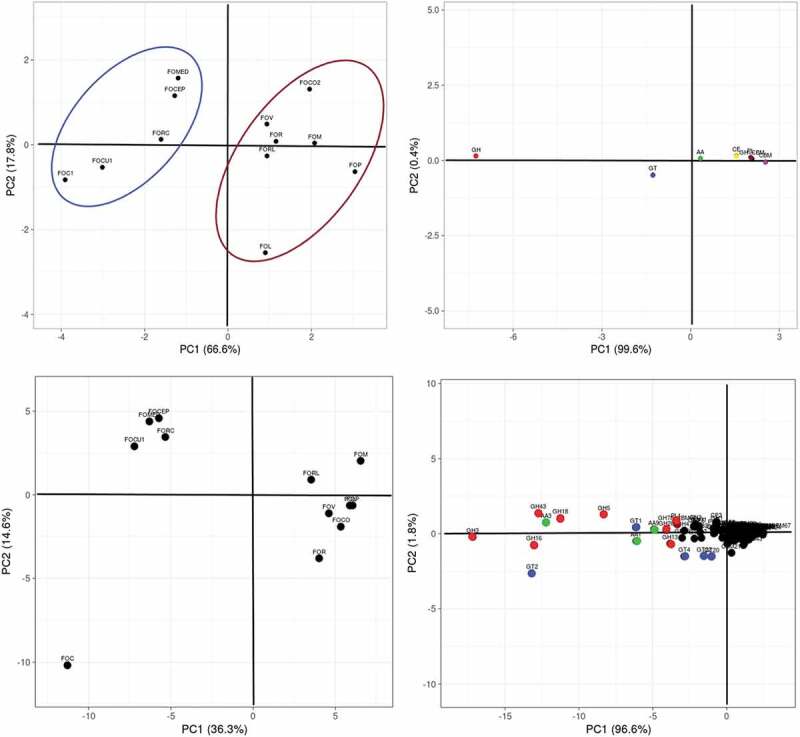
Principle component analysis of 12 ff. spp. A. based on number of CAZymes modules and B. based on copy number of CAZymes families of AA, CE, CBM, GH, GT, PL. (AA: Auxillary Activities, CE: Carbohydrate Esterase, CBM: Carbohydrate-Binding Module, GH: Glycosyl hydrolase, GT: Glycosyl Transferase, PL: Polysaccharide lyaseFOL: *F. oxysporum* f. sp. *lycopersici*, FORL*: F.oxysporum* f. sp. *radicis-lycopersici*, FOCO: *F. oxysporum* f. sp. *conglutinans*, FOR: *F. oxysporum* f. sp. *raphani*, FOV: *F. oxysporum* f.sp. *vasinfectum*, FOM: *F. oxysporum* f. sp. *melonis*, FOP: *F. oxysporum* f. sp. *pisi*, FOC: *F. oxysporum f*. sp. *ciceris*, FOCU: *F. oxysporum* f. sp. *cubense*, FORC: *F. oxysporum* f. sp. *radicis-cucmerinum*, FOMED: *F. oxysporum* f. sp. *medicaginis*, FOCEP*: F. oxysporum* f. sp. *cepae*)

In Principle Component Analysis, the variation based on the distribution of CAZymes modules was obtained which indicated PC1 to be 66.6% and PC2 to be 17.8% and enabled the ff. spp. to be clustered into two broad groups encompassing f. sp. *ciceris* (FOC), f. sp. *medicaginis* (FOMED), f. sp. *cubense* (FOCU), f. sp. *cepae* (FOCEP) and f. sp. *radicis-cucmerinum* (FORC) in one cluster while the rest including f. sp. *lycopersici* (FOL), f. sp. *pisi* (FOP), f. sp. *radicis-lycopersici* (FORL), f. sp. *raphani* (FOR), f. sp. *melonis* (FOM), f. sp. *vasinfectum* (FOV) and f. sp. *conglutinans* (FOCO) were distributed in another cluster (Figure 2A). Based on copy numbers of CAZymes families, PC1 represented 36.3% while PC2 represented 14.6% of variation (Figure 2B) which further outlined the FOC from the other ff. spp. clustered in a group (refer Figure 2A). The clustering was majorly influenced by the modules GH and GT followed by AA where the predominant families observed included GH3, GH5, GH13, GH16, GH18, GH43, GT1, GT2, GT4, AA1, AA3, AA9, and PL1.

These *formae speciales* are often considered as polyphyletic due to horizontal gene transfer (van Dam et al. 2018) which make them difficult to identify. Although variations observed in accessory genome also known as conditionally dispensable chromosomes (CDC) probably harbours effector genes responsible for host specificity (van Dam et al. 2016; Williams et al. 2016) despite the fact, the core genomes of many of the *formae speciales*, roughly of the same size (van Dam et al. 2016) consist majority of CAZymes. Hence, from the above evidences, it could be suggested that the CAZymes modules annotated in genomic assemblies of the ff. spp. might have a correlation established with the host specificity which may contribute to the closeness of FOC and FOMED but surprisingly, keeping FOP segregated even though it belongs to legumes infecting ff. sp. Also, FOC was further separated out from FOMED based on CAZymes families and this is also evidenced by (Williams et al. 2016), that these legumes infecting ff. spp. were rather sharing conserved candidate effector proteins and not large genomic region of pathogenicity-related content.

Interestingly, FORC affecting a range of cucurbits shows rot symptoms (Vakalounakis 1996; Vakalounakis et al. 2004; Vakalounakis and Fragkiadakis 1999) and since the symptom is similar to FOCEP (Entwistle 1990), both were clustered together. Therefore, based on the CAZymes distribution, these two legumes infecting ff. spp. (i.e. FOC and FOMED) were much closer to rot causing as well as monocot affecting ff. spp. than FOP which was clustered with other ff. spp. affecting host from the families of malvaceae, brassicaceae, solanaceae and cucurbitaceae.

Based on the information retrieved from the CAZy database, we limited our scope to families of GH, AA, CE, PL that are involved in plant cell wall degradation while focussing intensively on the secretory CAZYmes from the legumes infecting *formae speciales* and subsequent identification of secretory PCWDE.

### Distribution of Plant Cell Wall Degrading Enzymes (PCWDE) in legumes infecting formae speciales

Since these pathogens are necrotrophic in nature (Vajna 1985), the high copy number of PCWD CAZYmes encoded in the genome of the presently investigated organisms could be attributed to their mode of nutrition which is relatively recognised to be inevitable for their synthesis (Zhao et al. 2013). Thus, the overall distribution of the CAZymes modules constituting GH, AA, CE, and PL by FOC, FOMED and FOP accounted for 384, 449 and 514, respectively. Since these three ff. spp. correspond to infect legumes, their ability to degrade plant cell wall were analysed by sorting the CAZymes manually which are evidenced to be involved in catabolic processes like cellulose degradation, hemicellulose degradation, pectin degradation, lignin degradation and LPMO activities (Supplementary Table S4).

### Cellulose degrading

Accordingly, 16 cellulose-degrading CAZymes families were annotated in all the three legumes infecting ff. spp. with variation in their copy numbers which mostly constituted β-glucosidase (van den Brink and de Vries 2011), exo-endo- β- glucanase (Choi et al. 2013; Franková and Fry 2013; Rytioja et al. 2014). However, the high copy numbers of GH3 and GH5 corresponding to a β-glucosidase and an endo-β-1, 4-glucanase seemed to predominate with copy number ranging from 30 to 49 and 19 to 23, respectively. GH2 (β-galactosidase) and GH31 (α-glucosidase) exhibited moderate copy numbers ranging from 8 to 10 and GH1 (β-glucosidase) and GH17 (β-glucosidase) having 6–8 copy numbers. GH6 (endoglucanase), GH131 (exo/endoglucanase) and GH74 (endoglucanase) were present in single copy number in three of the legumes infecting ff. spp.

### Hemicellulose degrading

The number of families having hemicellulose degrading ability encoded in these legumes infecting ff. spp. were 26 and constituted xylanase, arabinofuranosidase, glucuronidase, acetyl xylan esterase, and xylosidase (Choi et al. 2013), belonging to both GH and CE families with abundant copy numbers of GH16, encoding for xyloglucantransferase and GH43 for β – xylosidase each having copy numbers ranging from 23 to 36 and 25 to 34, respectively. Beside GH families, the families CE3 (acetyl xylan esterase), CE4 (acetyl xylan esterase) and CE5 (cutinase) exhibited copy numbers between 8 and 11. GH62 (α-L-arabinofuranosidase), GH146 (chitosanase), GH10 (β-xylanase) associated with CBM1 (cellulose-binding function) and CE2 (acetyl xylan esterase) were present in single copy number in all of them.

### Pectin degrading

Here the families of CAZymes having pectin degrading ability constituted 22 families in these legumes infecting ff. spp. which mostly belonged to arabinase, rhamnogalacturonase, mannanase and galactase (Choi et al. 2013; Rytioja et al. 2014) constituting GH, CE and PL families, respectively. GH 43, an endo-α-1, 5-L-arabinanase was found to be higher in copy number ranging from 25 to 34. Subsequently, GH28 (poly galactouranase), GH47 (α-mannosidase) and GH76 (α-mannanase) with copy numbers varying from 9 to 13 were found in these legumes infecting ff. spp. PL1, a pectate lyase was higher in all of them and ranged between 11 and 12. In GH having CBM domain, GH78 (α-rhamnosidase) with CBM67 (L-rhamnose binding activity) was having copy number ranging from 8 to 14. The GH having single copy number in each of them was found to be GH38 (α-mannosidase). Whereas only carbohydrate esterase CE8 (pectin methylesterase) and CE12 (pectin acetyl esterase) families were present with copy number of four in each of them.

### Lignin-Degrading and Lytic Polysaccharide Mono-Oxygenase (LPMO)

Around 13 of the AA families were represented in these legumes infecting formae speciales with higher copy number in AA3 (25–36) which is a laccase, followed by AA1 (14–19), a cellubiose dehydrogenase; AA9 (13–17), an LPMO having cellulose cleaving activity and AA7 (6–11), a gluco-oligosaccharide oxidase. CAZymes belonging to AA module are involved in lignin degradation (Chen et al. 2012) and Copper (Cu) dependent LPMO (Busk and Lange 2015) which probably assist GH by cleaving with different substrates like cellulose (Beeson et al. 2015), hemicelluloses (Agger et al. 2014; Couturier et al. 2018) and starch (Vu et al. 2014; Vu and Marletta 2016). The families with single copy number were from AA13, a starch cleaving LPMO and AA14, a xylan cleaving LPMO, though AA4 (vanillyl-alcohol oxidase) and AA8 (iron reductase domain) were absent in FOMED.

Here the PCWDE families identified in the three legumes infecting ff. spp. were also reported in the studies done by (Chang et al. 2016; Ferreira Filho et al. 2017; Sista Kameshwar and Qin 2017). The families representative of GH94, GH130, PL10, which were supposed to be present in ascomycetes (Zhao et al. 2013) were found to be absent in these ff. spp. and our study also led to the observation of some more CAZymes families to be present in these ff. spp. involved in the process of cell-wall degradation. The families of GH which are degrading cellulose and hemicellulose identified with high copy numbers in these ff. spp. were also found to be higher in other species of ascomycetes belonging to both dothideomycetes and sordariomycetes (Zhao et al. 2013; Morales-Cruz et al. 2015; Paolinelli-Alfonso et al. 2016).

Accordingly, CAZymes families that were abundantly present (GH16, GH43, CE3, CE4 and CE5) evidenced their involvement in pathogenesis of *Fusarium* sp. (Kikot et al. 2009) and PL1 seemed to significantly contribute to virulence in pathogenesis (Kikot et al. 2009) Although both GH28 and GH47 are found in moderate copy numbers but might play a critical role in pectin degradation and degradation of both the hemicellulose and pectin, respectively (Zhao et al. 2013).

The high copy numbers of AA1, AA3 and AA9 in all these legumes infecting ff. spp. corresponded to lignin degradation and LPMO activities and perhaps the association of AA3 with AA9 was suggested to be involved in the stimulation of the lignocelluloses degradation (Langston et al. 2011). Also, the abundant copy number of CAZYmes families known to have a role in metabolic processes like cellulose, hemicellulose and pectin degradation in these ff. spp. as evidenced in *F. oxysporum* species by (Sista Kameshwar and Qin 2017) are known to deconstruct the plant cell wall. Hence, the analyses on plant cell wall degrading ability of these legumes infecting ff. spp. led us further to focus on secretory CAZymes. Therefore, these CAZymes belonging to modules GH, AA, CE and PL were assessed for their secretory nature.

### Identification and distribution of secretory CAZymes

All the pathogenic fungi produce a diverse array of secretory CAZymes, which are extracellular in nature and prominently contributes to phytopathogenic activity in the necrotrophic organisms irrespective of the fungal families. Hence, the analysis of the present study revealed nearly half of the CAZymes constituting GH, CE, PL and AA to encode for signal peptides. The signalP and DeepLoc analysis enabled not only to identify CAZymes encoding for signal peptides but also their corresponding subcellular locations in all the three legumes infecting ff. spp. Comparing the CAZymes coding for signal peptide revealed FOC and FOMED to represent approximately 52% while it was 45% in FOP. Further, subcellular localisations of these signal peptide coding CAZymes differentiated as extracellular and soluble were predicted to be high in FOC (84%) whereas around 50% were recognised in the other two legumes infecting ff. spp. (Table 1).Thus, subsequent embodiment of the modules representing the secretory CAZymes implies the families of AA9, CE5, GH5, GH16 and PL1 to be abundantly represented and exhibiting moderate representation of families AA1, AA5, CE3, CE4, CE16, GH3, GH18, GH28, GH43 and PL3 in all the three legumes infecting ff. spp. However, there was an overrepresentation of the families AA3, AA9, CE5, GH3, GH16, GH18, GH28, GH43 and PL4 in FOC (Table 2).

**Table 1.** Identification of CAZYmes coding for signal peptides and their sub-cellular localisation in legumes infecting *formae speciales*.

				EC (secretory)					
Organisms	Total CAZymes	CAzymes coding for signal peptide	% of CAZymes having signal peptide/Total CAZymes	GH	AA	CE	PL	Total	% of EC (secretory) CAZYmes/CAZymes with signal peptides
FOC	573	297	51.83	146	45	37	21	249	83.83
FOMED	653	337	51.60	93	22	24	22	161	47.77
FOP	826	376	45.52	110	27	24	23	184	48.93

FOC: f. sp. *ciceris*, FOMED: f. sp. *medicaginis*, Fop: f. sp. *pisi*, EC: Extracellular, SigP: Signal peptide, %: Percentage, GH: Glycosyl hydrolase, AA: Auxillary activity: CE: Carbohydrate esterase, PL: Polysaccharide lyase.

**Table 2.** Family-wise copy number of the predicted secretory CAzymes in three legumes infecting ff. spp.

Modules	Families	FOC	FOMED	FOP
Auxiliary activities	AA1	5	3	5
	AA2	1	0	0
	AA3	11	1	1
	AA5	5	4	5
	AA7	3	1	0
	AA8	1	1	1
	AA9	12	6	8
	AA11	4	2	2
	AA12	2	3	4
	AA13	0	0	1
	AA14	1	1	0
Carbohydrate esterase	CE1	2	4	4
	CE2	1	0	0
	CE3	7	3	3
	CE4	5	5	4
	CE5	10	7	7
	CE8	4	2	2
	CE12	4	0	1
	CE16	4	3	3
Glycosyl hydrolase	GH1	1	0	0
	GH2	1	0	0
	GH3	12	3	6
	GH5	8	11	11
	GH6	1	1	1
	GH7	3	3	6
	GH10	3	4	4
	GH11	3	3	4
	GH12	2	3	3
	GH13	1	1	1
	GH15	1	1	0
	GH16	10	7	8
	GH17	4	1	1
	GH18	14	4	6
	GH20	0	1	2
	GH24	1	2	1
	GH27	2	3	5
	GH28	10	7	8
	GH29	1	0	0
	GH30	1	2	1
	GH31	2	0	0
	GH32	2	1	2
	GH35	2	1	1
	GH37	1	0	0
	GH39	3	0	0
	GH43	18	9	8
	GH45	1	1	2
	GH49	1	1	1
	GH51	1	0	0
	GH53	1	0	0
	GH54	1	1	1
	GH55	2	3	2
	GH62	1	1	1
	GH64	3	0	4
	GH65	1	0	0
	GH67	1	2	2
	GH71	1	1	3
	GH74	1	1	1
	GH75	1	1	1
	GH76	1	0	0
	GH78	1	1	1
	GH79	2	0	0
	GH93	5	4	4
	GH105	1	0	0
	GH106	1	0	0
	GH114	2	2	2
	GH115	2	0	0
	GH125	0	1	0
	GH128	2	1	0
	GH131	1	1	1
	GH132	1	1	2
	GH139	1	0	1
	GH145	2	1	1
	GH146	1	1	1
Polysaccharide lyase	PL1	11	11	11
	PL3	5	7	7
	PL4	3	1	1
	PL9	2	2	2
	PL11	0	1	2

FOC: f. sp. *ciceris*, FOMED: f. sp. *medicaginis*, Fop: f. sp. *pisi*, GH: Glycosyl hydrolase, AA: Auxillary activity: CE: Carbohydrate esterase, PL: Polysaccharide lyase.

### Orthologues study of secretory CAZymes

The three legumes infecting ff. spp. formed 187 orthologous clusters with 49 clusters containing sequences from at least two of the ff. spp. and 121 clusters containing sequences from all the three ff. spp. (Supplementary Figure S1) in which 112 clusters contained single copy sequences from each organism.

The predicted secretory PCWDE protein sequences of FOC were involved in 162 clusters, FOP in 160 clusters and FOMED in 156 clusters with the number of sequences which did not form any clusters, i.e. singletons were 56, 10 and 8, respectively. Further analyses of the 121 clusters containing sequences from all the three ff. spp. revealed 90 clusters with GO annotations which were majorly involved in carbohydrate metabolic processes having hydrolase, lyase, carbohydrate and pectin binding activities (Supplementary Table S5). Out of these 90 clusters, 74 clusters were found to be extracellular according to assigned GO annotation process of which 12 clusters belonged to AA, 9 to CE, 16 to PL and 37 to GH and within the GH clusters, 4 clusters of GH were associated with CBM. Highest number of CAZYmes families involved in clusters were AA9 (6), CE5 (5), GH28 (6) and PL1 (10) (Table 3). Hence, these CAZYmes orthologues which were having maximum clusters might be suggested to play a role in survival and cell-wall degradation process as explored through evidences. Accordingly, AA9 family previously known as GH61 (Levasseur et al. 2013) present in high frequency of orthologue clusters and multiple copies in genome (Harris et al. 2010), assists GH and other families like PL and CE in the breakdown of cellulose (Monclaro and Filho 2017) whereas GH28 observed to play a significant role in pectin degradation is reported to involve in the process of plant cell wall degradation (García-Maceira *et al*., 2001; Sprockett et al. 2011). Moreover, the existence of CE5 corresponding to cutinase or acetyl xylan esterase (Kolattukudy 1981) and PL1, a pectate lyase (Herron et al. 2000), reported to be secreted by plant pathogens perhaps suggest to help in the process of cleaving cutin and pectin where both are crucial with one as protective covering and other as providing physical strength to the plants. Therefore, these abundant orthologue clusters are indeed crucial for the plant cell wall degradation process, identified in these legumes infecting ff. spp.

**Table 3.** Number of secretory CAZYmes families constituting orthologous clusters with their Uniprot and GO accessions.

CAZymes Modules	CAZyme families	No. of orthologue clusters	Function	Uniprot ID’s	GO annotation
AA	**AA1**	**3**	Laccase-2	P17489, Q96WM9	GO:0005576
	**AA3**	**1**	Cellobiose dehydrogenase	Q01738	GO:0005576
	**AA5**	**2**	Galactose oxidase	I1S2N3	GO:0005576
	**AA9**	**6**	Endoglucanase-4	O14405,A1DBS6,G2Q9T3, B0Y9G4, G2Q9T3	GO:0005576
CE	**CE1**	**2**	Feruloyl esterase B ,Probable acetylxylan esterase A	Q9HE18,B8NBI2	GO:0005576
	**CE5**	**5**	Acetylxylan esterase, Cutinase	Q99034,Q96UT0, Q99174,P11373	GO:0005576
	**CE8**	**2**	Pectinesterase	Q12535,P17872	GO:0005576
GH	**GH3**	**1**	Probable beta-glucosidase M	B8N5S6	GO:0005576
	**GH5**	**3**	Glucan endo-1,6-beta-glucosidase B , Endoglucanase 3, Mannan endo-1,4-beta-mannosidase B	Q5B6Q3,Q12624, Q5B833	GO:0005576
	**GH6**	**1**	Putative endoglucanase type B	P46236	GO:0005576
	**GH7**	**2**	Putative exoglucanase type C	P46238	GO:0005576
	**GH10+ CBM1**	**2**	Endo-1,4-beta-xylanase 1	P79046, P46239	GO:0005576
	**GH11**	**3**	Endo-1,4-beta-xylanase A	Q92245,I1S2K3,I1RII8	GO:0005576
	**GH12**	**1**	Probable xyloglucan-specific endo-beta-1,4-glucanase A	A1D4F1	GO:0005576
	**GH17**	**1**	Glucan 1,3-beta-glucosidase	O13990	GO:0005576
	**GH18**	**1**	Endochitinase B1	Q873X9	GO:0005576
	**GH27**	**2**	Alpha-galactosidase	Q99172,O94221	GO:0005576
	**GH28**	**6**	Probable exopolygalacturonase B ,Probable rhamnogalacturonase E ,Polygalacturonase, Endopolygalacturonase 1 ,Probable endopolygalacturonase ,Endo-xylogalacturonan hydrolase A	B0YAA4,Q1ZZM3, Q07181,Q00446, A1D145,Q9UUZ2	GO:0005576
	**GH32**	**1**	Extracellular endo-inulinaseinuA	O74641	GO:0005576
	**GH35**	**1**	Probable beta-galactosidase A	Q700S9	GO:0005576
	**GH43**	**3**	Probable arabinan endo-1,5-alpha-L-arabinosidase C, Arabinan endo-1,5-alpha-L-arabinosidase B, Probable arabinan endo-1,5-alpha-L-arabinosidase A	A5AAG2, Q5AZC8, A1D5W1	GO:0005576
	**GH45**	**1**	Putative endoglucanase type K	P45699	GO:0005576
	**GH49**	**1**	Dextranase	P48845	GO:0005576
	**GH54+ CBM24**	**1**	Probable alpha-L-arabinofuranosidase B	Q4WL66	GO:0005576
	**GH62**	**1**	Probable alpha-L-arabinofuranosidaseaxhA	Q2U7D2	GO:0005576
	**GH67**	**1**	Alpha-glucuronidase	Q99024	GO:0005576
	**GH71+ CBM24**	**1**	Glucan endo-1,3-alpha-glucosidase agn1	O13716	GO:0005576
	**GH74**	**1**	Xyloglucanase	Q7Z9M8	GO:0005576
	**GH75**	**1**	Endo-chitosanase	Q7Z9M8	GO:0005576
	**GH132**	**1**	Extracellular endo-inulinase	Q4WGL5	GO:0005576
PL	**PL1**	**10**	Pectin lyase, A,B,C,F	Q5AVN4, B0XMA2, Q0CZD4, Q2UCT7, Q00374, Q4WV10, Q00374, Q00645, O59939	GO:0005576
	**PL3**	**5**	Probable pectatelyase D,E,F,H	Q5B024, A1C4B8, Q0CJ49, Q5ATC7	GO:0005576
	**PL4**	**1**	Probable rhamnogalacturonatelyase A	Q4W9T6	GO:0005576

GH: Glycosyl hydrolase, AA: Auxillary activity: CE: Carbohydrate esterase, PL: Polysaccharide lyase; GO: 0005576, extracellular.

## Differential gene expression analysis of secretory CAZymes

**Figure 3.**
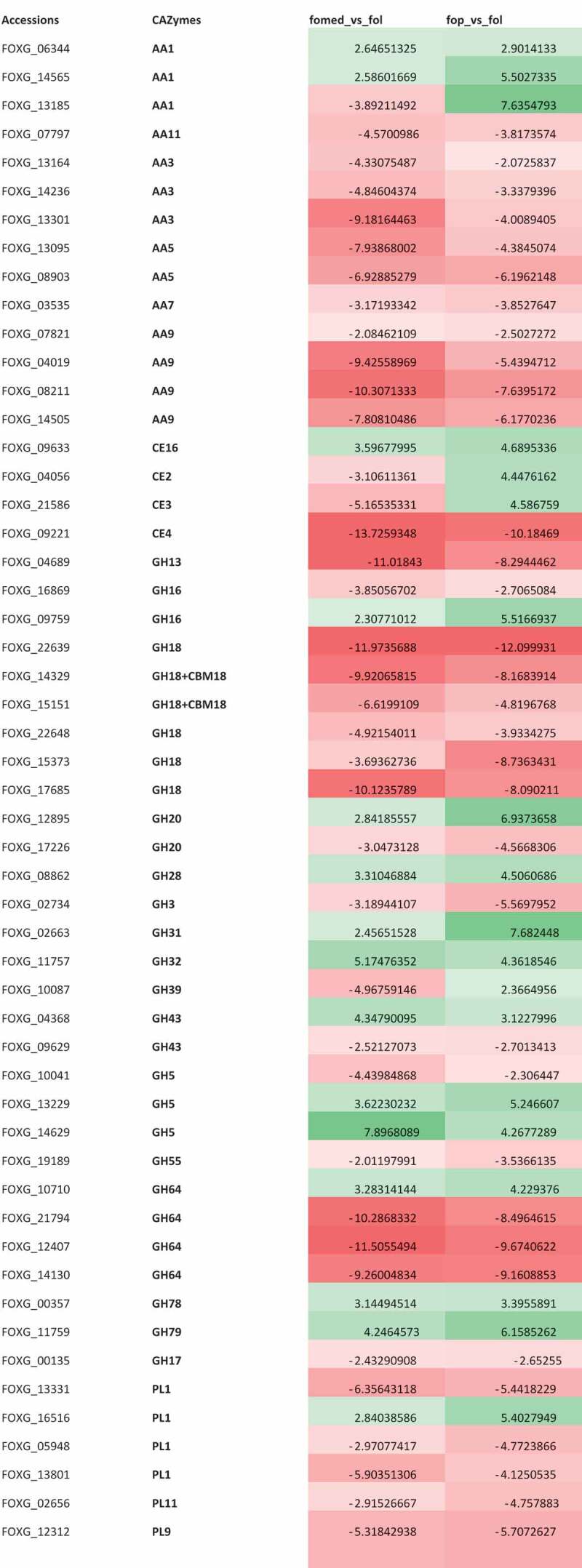
Differential expression heatmap of the genes coding for extra cellular CAZymes commonly present in the two comparisons (FOMED: f. sp. *medicaginis*, FOP: f. sp. *pisi*, FOL: f. sp. *lycopersici*).(GH: Glycosyl hydrolase, AA: Auxillary activity: Ce: Carbohydrate esterase, PL: Polysaccharide lyase).

The differential expression analysis after FDR cut-off of <0.05 and LogFC of ≥ |2|, resulted in 5483 significant differentially expressed genes (DEGs) for FOMED vs. FOL and 5599 for FOP vs. FOL where 3525 DEG accessions were identified to be common in both the studies through Venn diagram (Supplementary Figure S2). These 3525 common DEGs when subjected to CAZymes annotation resulted in 109 CAZymes which were both down- and up-regulated. In order to identify families belonging to modules of AA, GH, CE, and PL that perhaps encodes for secretory peptides, accounted for 87 differentially expressed (DE) CAZymes (Supplementary Table S6), from which 56 were observed to code for secretory peptides, out of which 53 were identified as extracellular CAZymes belonging to the families of AA, GH, CE and PL while the other 3 were found to be localised in cell membrane and lysozyme (Supplementary S7). The pattern of expression in both the legumes infecting ff. spp. were almost similar except for CE2, CE3 and one accession coding for AA1 which showed contrasting expression pattern in both the *formae speciales*. Most of the secretory CAZymes categorised as PCWDE were significantly down regulated with exceptional accessions coding for the same CAZymes families such as GH16, GH43, and PL1 which were found to be both up- and down-regulated in these ff. spp. and the contrasting difference might have been contributed by the nutritional media in which they were grown. The accessions coding for families like CE16 (acetyl esterase), GH28 (polygalacturonase) and GH78 (α-L- rhamnosidase) were found to be up regulated in both the ff. spp. and are involved in hemicellulose and pectin degradation (Figure 3).

The observation on the majority of secretory CAZymes annotated revealed GH5, GH28, AA9 and PL1 to be abundantly present in the genomic assemblies as well as in orthologue clusters in the three of the legumes infecting *formae speciales* and were also found to be differentially expressed. The observation conceived from the secretory CAZymes significantly expressed are in accordance with the study done by Zhang et al. (2018) who revealed a transcription factor was controlling the expression of genes coding for GH3, GH5, GH28, AA3, AA9 and PL1 in *Verticillium dahlia*, a soil-borne wilt causing pathogen of ascomycetes, although the families, GH5, an endoglucanase was having a mixed expression and GH28, a polygalactouranase was significantly up regulated in these two legumes infecting ff. spp. involving in degradation of cellulose and pectin, respectively. This could be further substantiated by a study done on *Aspergillus niger* (Daly et al. 2017), where genes coding for pectin lyase induced by galacturonic acid and endoglucanase were found to be significantly up-regulated when grown on wheat straw. In general, the secretory CAZymes identified in higher and moderate copy numbers were found to be differentially expressed in these *formae speciales* and interestingly, the accessions coding for pectin degrading CAZYmes were up-regulated implicating their importance in growth and pathogenesis. Hence, the family GH28 with other less abundant pectin degrading families are found to be up-regulated and could possibly play role in pathogenesis.

## Conclusion

Genome-wide distribution of CAZymes families among the ff. spp. revealed the variations in the copy number of the families and also the abundance of some PCWDE in the legumes infecting ff. spp. Thus, the details generated through the present study significantly contributed to the distribution pattern of these important PCWDE families and led us to the identification and annotation of some of the key families which are involved in cell-wall degradation process among the legumes infecting ff. spp. PCWDE and their orthologues especially present in single copy in these legumes infecting ff. spp. and also differential expression analysis shed light on some of the secretory CAZymes with their expressional changes. Therefore, the important PCWDE orthologues identified through this study in these ff. spp. which were also abundantly present in the genomic assemblies and also found to be significantly expressed may further enable us to choose these candidate CAZymes to understand their co-ordination, mechanistic role and explore them as targets to attenuate these phytopathogens.

## References

[CIT0001] Agger JW, Isaksen T, Várnai A, Vidal-Melgosa S, Willats WG, Ludwig R, Horn SJ, Eijsink VG. 2014. Discovery of LPMO activity on hemicelluloses shows the importance of oxidative processes in plant cell wall degradation. Proc Natl Acad Sci. 111(17):6287–6292. doi:10.1073/pnas.1323629111. 24733907 PMC4035949

[CIT0002] Almagro Armenteros JJ, Sønderby CK, Sønderby SK, Nielsen H, Winther O. 2017. DeepLoc: prediction of protein subcellular localization using deep learning. Bioinf. 33(21):3387–3395. Edited by J. Hancock. Oxford University Press. doi:10.1093/bioinformatics/btx431. 29036616

[CIT0003] Beeson WT, Vu VV, Span EA, Phillips CM, Marletta MA. 2015. Cellulose degradation by polysaccharide monooxygenases. Annu Rev Biochem. 84:923–946. doi:10.1146/annurev-biochem-060614-034439 25784051

[CIT0004] Berlemont R. 2017. Distribution and diversity of enzymes for polysaccharide degradation in fungi. Sci Rep. 7(1):222. doi:10.1038/s41598-017-00258-w. 28302998 PMC5428031

[CIT0005] Blackman LM, Cullerne DP, Hardham AR. 2014. ‘Bioinformatic characterisation of genes encoding cell wall degrading enzymes in the phytophthora parasitica genome.’, *BMC genomics*. BioMed Central. 15:785. doi:10.1186/1471-2164-15-785 PMC4176579 25214042

[CIT0006] Boraston AB, Bolam DN, Gilbert HJ, Davies GJ. 2004. Carbohydrate-binding modules: fine-tuning polysaccharide recognition. Biochem J. 382(Pt 3):769–781. doi:10.1042/BJ20040892. 15214846 PMC1133952

[CIT0007] Buchfink B, Xie C, Huson DH. 2015. ‘Fast and sensitive protein alignment using DIAMOND’, *nature methods*. Nature Publishing Group. 12(1):59–60. doi:10.1038/nmeth.3176. 25402007

[CIT0008] Busk PK, Lange L. 2015. ‘Classification of fungal and bacterial lytic polysaccharide monooxygenases’, *BMC Genomics*. BioMed Central. 16(1):368. doi:10.1186/s12864-015-1601-6. PMC4424831 25956378

[CIT0009] Busk PK, Pilgaard B, Lezyk MJ, Meyer AS, Lange L. 2017. ‘Homology to peptide pattern for annotation of carbohydrate-active enzymes and prediction of function’, *BMC bioinformatics*. BioMed Central. 18(1):214. doi:10.1186/s12859-017-1625-9. PMC5389127 28403817

[CIT0010] Campbell JA, Davies GJ, Bulone V, Henrissat B. A. 1997. A classification of nucleotide-diphospho-sugar glycosyltransferases based on amino acid sequence similarities. Biochem J. 326(Pt 3):929–939. http://www.ncbi.nlm.nih.gov/pubmed/9334165 9334165 10.1042/bj3260929u PMC1218753

[CIT0011] Caracuel Z, Martínez-Rocha AL, Di Pietro A, Madrid MP, Roncero MI. 2005. *Fusarium oxysporum gas1* encodes a putative β-1, 3-glucanosyltransferase required for virulence on tomato plants. Mol Plant-Microbe Interact. 18(11):1140–1147. doi:10.1094/MPMI-18-1140. 16353549

[CIT0012] Chang H-X, Yendrek CR, Caetano-Anolles G, Hartman GL. 2016. ‘Genomic characterization of plant cell wall degrading enzymes and in silico analysis of xylanses and polygalacturonases of fusarium virguliforme’, *BMC microbiology*. BioMed Central. 16(1):147. doi:10.1186/s12866-016-0761-0. PMC4941037 27405320

[CIT0013] Chen Y, Sarkanen S, Wang -Y-Y. 2012. ‘Lignin-degrading enzyme activities’, in *biomass conversion*. Totowa (NJ): Humana Press; p. 251–268. doi:10.1007/978-1-61779-956-3_21 22843404

[CIT0014] Choi J, Kim KT, Jeon J, Lee YH. 2013. Fungal plant cell wall-degrading enzyme database: a platform for comparative and evolutionary genomics in fungi and oomycetes.’, *BMC genomics*. BioMed Central. 14(Suppl 5 (Suppl5)):S7. doi:10.1186/1471-2164-14-S5-S7. PMC3852112 24564786

[CIT0015] Correll JC. 1991. The relationship between formae speciales, races, and vegetative compatibility groups in fusarium oxysporum. Phytopatholo. 81:1061–1064. https://www.apsnet.org/publications/phytopathology/backissues/Documents/1991Articles/phyto81n09_1061.pdf.

[CIT0016] Courtade G, Wimmer R, Røhr ÅK, Preims M, Felice AKG, Dimarogona M, Vaaje-Kolstad G, Sørlie M, Sandgren M, Ludwig R, *et al*. 2016. ‘Interactions of a fungal lytic polysaccharide monooxygenase with β-glucan substrates and cellobiose dehydrogenase’. Proc Natl Acad Sci. 113(21):5922–5927. National Academy of Sciences. doi:10.1073/pnas.1602566113. 27152023 PMC4889390

[CIT0017] Coutinho PM, Deleury E, Davies GJ, Henrissat B. 2003. An evolving hierarchical family classification for glycosyltransferases. J Mol Biol. 328(2):307–317. http://www.ncbi.nlm.nih.gov/pubmed/12691742 12691742 10.1016/s0022-2836(03)00307-3

[CIT0018] Couturier M, Ladeveze S, Sulzenbacher G, Ciano L, Fanuel M, Moreau C, Villares A, Cathala B, Chaspoul F, Frandsen KE, Labourel A. 2018. ‘Lytic xylan oxidases from wood-decay fungi unlock biomass degradation’. Nat Chem Biol. 14(3):306–310. Nature Publishing Group. doi:10.1038/nchembio.2558. 29377002

[CIT0019] Daly P, Van Munster JM, Blythe MJ, Ibbett R, Kokolski M, Gaddipati S, Lindquist E, Singan VR, Barry KW, Lipzen A, Ngan CY. 2017. ‘Expression of aspergillus niger CAZymes is determined by compositional changes in wheat straw generated by hydrothermal or ionic liquid pretreatments’, *biotechnology for biofuels*. BioMed Central. 10:35. doi:10.1186/S13068-017-0700-9 PMC5294722 28184248

[CIT0020] Davies GJ, Gloster TM, Henrissat B. 2005. ‘Recent structural insights into the expanding world of carbohydrate-active enzymes’. Curr Opin Struct Biol. . 15(6):637–645. Elsevier Current Trendsdoi:10.1016/J.SBI.2005.10.008. 16263268

[CIT0021] de Hoon MJL, Imoto S, Nolan J, Miyano S. 2004. Open source clustering software. Bioinf. 20(9):1453–1454. Narnia. doi:10.1093/bioinformatics/bth078. 14871861

[CIT0022] Del Mar Jiménez-Gasco M, Pérez-Artés E, Jiménez-Diaz RM. 2001. Identification of pathogenic races 0, 1B/C, 5, and 6 of fusarium oxysporum F. Sp. Ciceris with Random Amplified Polymorphic DNA (RAPD). Eur J Plant Pathol. 107(2):237–248. Kluwer Academic Publishers. doi:10.1023/A:1011294204630.

[CIT0023] Entwistle AR. 1990. ‘Root diseases in onions and allied crops’, in *root diseases, in onions and allied crops: volume II — agronomy, biotic actions, pathology, and crop protection, (eds H.D. Rabinowitch and J.L. Brewster)*. Boca Raton (Florida): CRC Press; p. 103–154.

[CIT0024] Essarioui A, Mokrini F, Afechtal M. 2016. Molecular interactions between tomato and its wilt pathogen fusarium oxysporum f. sp. lycopersici- a review. Revue Marocaine Des Sciences Agronomiques Et Vétérinaires. 4:66–74.

[CIT0025] Ferreira Filho JA, Horta MA, Beloti LL, dos Santos CA, de Souza AP. 2017. Carbohydrate-active enzymes in trichoderma harzianum: a bioinformatic analysis bioprospecting for key enzymes for the biofuels industry. BMC Genomics. 18(1):1–12. doi:10.1186/s12864-017-4181-9. 29025413 PMC5639747

[CIT0026] Finn RD, Clements J, Eddy SR. 2011. ‘HMMER web server: interactive sequence similarity searching’. Nucleic Acids Res. 39(suppl):W29–W37. Oxford University Press. doi:10.1093/nar/gkr367. 21593126 PMC3125773

[CIT0027] Franková L, Fry SC. 2013. Biochemistry and physiological roles of enzymes that “cut and paste” plant cell-wall polysaccharides. J Exp Bot. 64(12):3519–3550. doi:10.1093/jxb/ert201. 23956409

[CIT0028] García-Maceira FI, Di Pietro A, Huertas-González MD, Ruiz-Roldán MC, Roncero MIG. 2001. Molecular characterization of an Endopolygalacturonase from *Fusarium oxysporum* expressed during early stages of infection. *Applied and Environmental Microbiology*. 67(5):67.5.2191–2196.2001. 10.1128/AEM.67.5.2191-2196.2001 PMC92854 11319099

[CIT0029] Garron M-L, Cygler M. 2010. ‘Structural and mechanistic classification of uronic acid-containing polysaccharide lyases’. *Glycobiology*. Narnia. 20(12):1547–1573. doi:10.1093/glycob/cwq122. 20805221

[CIT0030] Giardine B, Riemer C, Hardison RC, Burhans R, Elnitski L, Shah P, Zhang Y, Blankenberg D, Albert I. 2005. Galaxy: a platform for interactive large-scale genome analysis. Genome Res. 15(10):1451–1455. Cold Spring Harbor Laboratory Press. doi:10.1101/GR.4086505. 16169926 PMC1240089

[CIT0031] Gibson DM, King BC, Hayes ML, Bergstrom GC. 2011. Plant pathogens as a source of diverse enzymes for lignocellulose digestion. Curr Opin Microbiol. 14(3):264–270. Elsevier Current Trends. doi:10.1016/J.MIB.2011.04.002. 21536481

[CIT0032] Glass NL, Schmoll M, Cate JH, Coradetti S. 2013. ‘Plant cell wall deconstruction by ascomycete fungi’, *annual review of microbiology*. Annu Rev. 67(1):477–498. doi:10.1146/annurev-micro-092611-150044. 23808333

[CIT0033] Grigoriev IV, Nikitin R, Haridas S, Kuo A, Ohm R, Otillar R, Riley R, Salamov A, Zhao X, Korzeniewski F, *et al*. 2014. MycoCosm portal: gearing up for 1000 fungal genomes. Nucleic Acids Res. 42(D1):D699–D704. Oxford University Press. doi:10.1093/nar/gkt1183. 24297253 PMC3965089

[CIT0034] Harris PV, Welner D, McFarland KC, Re E, Navarro Poulsen JC, Brown K, Salbo R, Ding H, Vlasenko E. 2010. ‘Stimulation of lignocellulosic biomass hydrolysis by proteins of glycoside hydrolase family 61: structure and function of a large, enigmatic family’, *biochemistry*. Am Chem Soc. 49(15):3305–3316. doi:10.1021/bi100009p. 20230050

[CIT0035] Haware MP, Nene YL, Mathur SB. 1986. Seed borne diseases of chickpea. Technical Bulletin 1, Danish government institute of Se ed technology for Developing Countries, Copenhagen, pp. 1–32.

[CIT0036] Henrissat B. 1991. A classification of glycosyl hydrolases based on amino acid sequence similarities. Biochem J. 280((Pt 2)(2)):309–316. Portland Press Limited. doi:10.1042/BJ2800309. 1747104 PMC1130547

[CIT0037] Henrissat B, Bairoch A. 1993. New families in the classification of glycosyl hydrolases based on amino acid sequence similarities. Biochem J. 293(Pt 3):781–788. http://www.ncbi.nlm.nih.gov/pubmed/8352747. 8352747 10.1042/bj2930781 PMC1134435

[CIT0038] Henrissat B, Davies G. 1997. Structural and sequence-based classification of glycoside hydrolases. Curr Opin Struct Biol. 7(5):637–644. http://www.ncbi.nlm.nih.gov/pubmed/9345621. 9345621 10.1016/s0959-440x(97)80072-3

[CIT0039] Hepple S. 1963. Infection of pea plants by Fusarium oxysporum f.sp. pisi in naturally infested soil. Trans Br Mycol Soc. 46(4):585–594. Elsevier. doi:10.1016/S0007-1536(63)80060-1.

[CIT0040] Herron SR, Benen JA, Scavetta RD, Visser J, Jurnak F. 2000. Structure and function of pectic enzymes: virulence factors of plant pathogens. Proc Natl Acad Sci U S A. 97(16):8762–8769. National Academy of Sciences. http://www.ncbi.nlm.nih.gov/pubmed/10922032 10922032 10.1073/pnas.97.16.8762 PMC34009

[CIT0041] Hoff KJ, Stanke M. 2013. WebAUGUSTUS–a web service for training AUGUSTUS and predicting genes in eukaryotes. Nucleic Acids Res. 41(W1):W123–W128. Oxford University Press. doi:10.1093/nar/gkt418. 23700307 PMC3692069

[CIT0042] Infantino A, Kharrat M, Riccioni L, Coyne CJ, McPhee KE, Grünwald NJ. 2006. Screening techniques and sources of resistance to root diseases in cool season food legumes. Euphytica. 147(1–2):201–221. Kluwer Academic Publishers. doi:10.1007/s10681-006-6963-z.

[CIT0043] Jorge I, Navas-Cortes JA, Jimenez-Diaz RM, Tena M. 2006. Cell wall degrading enzymes in fusarium wilt of chickpea: correlation between pectinase and xylanase activities and disease development in plants infected with two pathogenic races of fusarium oxysporum f. sp. ciceris ’. Can J Bot. 84(9):1395–1404. doi:10.1139/b06-103.

[CIT0044] Kikot GE, Hours RA, Alconada TM. 2009. Contribution of cell wall degrading enzymes to pathogenesis of *Fusarium graminearum* : a review. J Basic Microbiol. 49(3):231–241. John Wiley & Sons, Ltd. doi:10.1002/jobm.200800231. 19025875

[CIT0045] Kim D, Langmead B, Salzberg SL. 2015. HISAT: a fast spliced aligner with low memory requirements. Nat Methods. 12(4):357–360. Nature Publishing Group. doi:10.1038/nmeth.3317. 25751142 PMC4655817

[CIT0046] Kolattukudy PE. 1981. Structure, biosynthesis, and biodegradation of cutin and suberin. Annu Rev Plant Physiol. 32(1):539–567. Annual Reviews 4139 El Camino Way, P.O. Box 10139, Palo Alto, CA 94303-0139, USA. doi:10.1146/annurev.pp.32.060181.002543.

[CIT0047] Kubicek CP, Starr TL, Glass NL. 2014. ‘Plant cell wall–degrading enzymes and their secretion in plant-pathogenic fungi’, *annual review of phytopathology*. Annu Rev. 52(1):427–451. doi:10.1146/annurev-phyto-102313-045831. 25001456

[CIT0048] Langston JA, Shaghasi T, Abbate E, Xu F, Vlasenko E Sweeney MD. 2011. ‘Oxidoreductive cellulose depolymerization by the enzymes cellobiose dehydrogenase and glycoside hydrolase 61.’, *applied and environmental microbiology*. Am Soc Microbiol. 77(19):7007–7015. doi:10.1128/AEM.05815-11. PMC3187118 21821740

[CIT0049] Leslie JF, Summerell BA. 2006. The fusarium laboratory manual. Blackwell Pub. ISBN: 978-0-813-81919-8. https://www.wiley.com/en-us/The+Fusarium+Laboratory+Manual-p-9780813819198

[CIT0050] Levasseur A, Drula E, Lombard V, Coutinho PM, Henrissat B. 2013. ‘Expansion of the enzymatic repertoire of the CAZy database to integrate auxiliary redox enzymes’, *biotechnology for biofuels*. BioMed Central. 6(1):41. doi:10.1186/1754-6834-6-41. PMC3620520 23514094

[CIT0051] Liao Y, Smyth GK, Shi W. 2014. featureCounts: an efficient general purpose program for assigning sequence reads to genomic features. Bioinf. 30(7):923–930. Narnia. doi:10.1093/bioinformatics/btt656. 24227677

[CIT0052] Lombard V, Bernard T, Rancurel C, Brumer H, Coutinho PM, Henrissat B. 2010. A hierarchical classification of polysaccharide lyases for glycogenomics. Biochem J. 432(3):437–444. doi:10.1042/BJ20101185. 20925655

[CIT0053] Lombard V, Golaconda Ramulu H, Drula E, Coutinho PM, Henrissat B. 2014. The carbohydrate-active enzymes database (CAZy) in 2013. Nucleic Acids Res. 42(Database issue):D490–5. England. doi:10.1093/nar/gkt1178. 24270786 PMC3965031

[CIT0054] Love MI, Huber W, Anders S. 2014. ‘Moderated estimation of fold change and dispersion for RNA-seq data with DESeq2ʹ, *genome biology*. BioMed Central. 15(12):550. doi:10.1186/s13059-014-0550-8. PMC4302049 25516281

[CIT0055] Metsalu T, Vilo J. 2015. ClustVis: a web tool for visualizing clustering of multivariate data using principal component analysis and heatmap. Nucleic Acids Res. 43(W1):W566–W570. Oxford University Press. doi:10.1093/nar/gkv468. 25969447 PMC4489295

[CIT0056] Monclaro AV, Filho EXF. 2017. Fungal lytic polysaccharide monooxygenases from family AA9: recent developments and application in lignocellulose breakdown. Int J Biol Macromol. 102:771–778. doi:10.1016/j.ijbiomac.2017.04.077 28450248

[CIT0057] Morales-Cruz A, Amrine KC, Blanco-Ulate B, Lawrence DP, Travadon R, Rolshausen PE, Baumgartner K, Cantu D. 2015. ‘Distinctive expansion of gene families associated with plant cell wall degradation, secondary metabolism, and nutrient uptake in the genomes of grapevine trunk pathogens’, *BMC genomics*. BioMed Central. 16(1):469. doi:10.1186/s12864-015-1624-z. PMC4472170 26084502

[CIT0058] Nene YL, Reddy MV, Haware MP, Ghanekar AM, Amin KS, Pande S, Sharma M. 2012. Field diagnosis of chickpea. Information Bulletin No. 28 (revised). Patancheru, A.P. 502 324, India: International Crops Research Institute for the Semi-Arid Tropics. 60 pp. ISBN 92-9066-199-2. http://oar.icrisat.org/6601/1/InfoBulletin_28-ICRISAT_2012.pdf

[CIT0059] Nielsen H. 2017. Predicting secretory proteins with signalP. New York (NY): Humana Press; p. 59–73. doi:10.1007/978-1-4939-7015-5_6 28451972

[CIT0060] Paolinelli-Alfonso M, Villalobos-Escobedo JM, Rolshausen P, Herrera-Estrella A, Galindo-Sánchez C, López-Hernández JF, Hernandez-Martinez R. 2016. ‘Global transcriptional analysis suggests Lasiodiplodia theobromae pathogenicity factors involved in modulation of grapevine defensive response’, *BMC genomics*. BioMed Central. 17(1):615. doi:10.1186/s12864-016-2952-3. PMC4981995 27514986

[CIT0061] Rhodes LH. 2015. Front matter. In: Samac DA, Rhodes LH, Lamp WO editors. Compendium of alfalfa diseases and pests. 3rd ed. St. Paul, MN: The American Phytopathological Society; p.i–vi. doi:10.1094/9780890544488.fm.

[CIT0062] Rytioja J, Hildén K, Yuzon J, Hatakka A, de Vries RP, Mäkelä MR. 2014. ‘Plant-polysaccharide-degrading enzymes from basidiomycetes.’, *microbiology and molecular biology reviews : MMBR*. Am Soc Microbiol. 78(4):614–649. doi:10.1128/MMBR.00035-14. PMC4248655 25428937

[CIT0063] Singh RK, Hasan A, Chaudhary RG. 2010. Variability in *Fusarium oxysporum* f. sp. *ciceri* causing vascular wilt in chickpea. Arch Phytopathol Plant Prot. 43(10):987–995. Taylor & Francis. doi:10.1080/03235400802214836.

[CIT0064] Sista Kameshwar AK, Qin W. 2017. Comparative study of genome-wide plant biomass-degrading CAZymes in white rot, brown rot and soft rot fungi. Mycol. 1–13. Taylor & Francis. doi:10.1080/21501203.2017.1419296. PMC6059041 30123665

[CIT0065] Sprockett DD, Piontkivska H, Blackwood CB. 2011. Evolutionary analysis of glycosyl hydrolase family 28 (GH28) suggests lineage-specific expansions in necrotrophic fungal pathogens. Gene. 479(1–2):29–36. Elsevier. doi:10.1016/J.GENE.2011.02.009. 21354463

[CIT0066] Takken F, Rep M. 2010. The arms race between tomato and *Fusarium oxysporum*. Mol Plant Pathol. 11(2):309–314. John Wiley & Sons, Ltd (10.1111). doi:10.1111/j.1364-3703.2009.00605.x. 20447279 PMC6640361

[CIT0067] Vajna L. 1985. Phytopathogenic fusarium oxysporum schlecht, as a necrotrophic mycoparasite. J Phytopathol. 114(4):338–347. John Wiley & Sons, Ltd (10.1111). doi:10.1111/j.1439-0434.1985.tb00629.x.

[CIT0068] Vakalounakis DJ. 1996. Root and stem rot of cucumber caused by *fusarium oxysporum* f. sp. *radicis-cucumerinum* f. sp. nov. Plant Dis. 80(3):313. doi:10.1094/PD-80-0313. 30818711

[CIT0069] Vakalounakis DJ, Fragkiadakis GA. 1999. ‘Genetic diversity of *fusarium oxysporum* isolates from cucumber: differentiation by pathogenicity, vegetative compatibility, and RAPD fingerprinting’, *phytopathology*. Am Phytopathological Soc. 89(2):161–168. doi:10.1094/PHYTO.1999.89.2.161. 18944791

[CIT0070] Vakalounakis DJ, Wang Z, Fragkiadakis GA, Skaracis GN, Li DB. 2004. ‘Characterization of *fusarium oxysporum* isolates obtained from cucumber in China by pathogenicity, VCG, and RAPD’, *plant disease*. Am Phytopathological Soc. 88(6):645–649. doi:10.1094/PDIS.2004.88.6.645. 30812586

[CIT0071] van Dam P, de Sain M, ter Horst A, van der Gragt M. 2018. ‘Use of comparative genomics-based markers for discrimination of host specificity in fusarium oxysporum.’, *Applied and environmental microbiology*. Am Soc Microbiol. 84(1):e01868–17. doi:10.1128/AEM.01868-17. PMC5734036 29030446

[CIT0072] van Dam P, Fokkens L, Schmidt SM, Linmans JH, Kistler HC. 2016. Effector profiles distinguish *formae speciales* of *fusarium oxysporum*. Environ Microbiol. 18(11):4087–4102. John Wiley & Sons, Ltd (10.1111). doi:10.1111/1462-2920.13445. 27387256

[CIT0073] van den Brink J, de Vries RP. 2011. Fungal enzyme sets for plant polysaccharide degradation. Appl Microbiol Biotechnol. 91(6):1477. doi:10.1007/s00253-011-3473-2. 21785931 PMC3160556

[CIT0074] Vu VV, Beeson WT, Span EA, Farquhar ER, Marletta MA. 2014. ‘A family of starch-active polysaccharide monooxygenases’. Proc Natl Acad Sci U.S.A. 111(38):13822–13827. National Academy of Sciences. doi:10.1073/pnas.1408090111. 25201969 PMC4183312

[CIT0075] Vu VV, Marletta MA. 2016. Starch-degrading polysaccharide monooxygenases. Cell Mol Life Sci. 73:2809–2819. doi:10.1007/s00018-016-2251-9 PMC11108391 27170366

[CIT0076] Wang Y, Coleman-Derr D, Chen G, Gu YQ. 2015. OrthoVenn: a web server for genome wide comparison and annotation of orthologous clusters across multiple species. Nucleic Acids Res. 43(W1):W78–84. Oxford University Press. doi:10.1093/nar/gkv487. 25964301 PMC4489293

[CIT0077] Williams AH, Sharma M, Thatcher LF, Azam S, Hane JK, Sperschneider J, Kidd BN, Anderson JP, Ghosh R, Garg G, Lichtenzveig J. 2016. ‘Comparative genomics and prediction of conditionally dispensable sequences in legume-infecting fusarium oxysporum formae speciales facilitates identification of candidate effectors.’, *BMC genomics*. BioMed Central. 17:191. doi:10.1186/s12864-016-2486-8 PMC4779268 26945779

[CIT0078] Zhang H, Yohe T, Huang L, Entwistle S, Wu P, Yang Z, Busk PK, Xu Y, Yin Y. 2018. dbCAN2: a meta server for automated carbohydrate-active enzyme annotation. Nucleic Acids Res. 46(W1):W95–W101. Oxford University Press. doi:10.1093/nar/gky418. 29771380 PMC6031026

[CIT0079] Zhang W-Q, Gui Y-J, Short DPG, Li T-G, Zhang -D-D, Zhou L, Liu C, Bao Y-M, Subbarao KV, Chen J-Y, *et al*. 2018. *Verticillium dahliae* transcription factor VdFTF1 regulates the expression of multiple secreted virulence factors and is required for full virulence in cotton. Mol Plant Pathol. 19(4):841–857. John Wiley & Sons, Ltd (10.1111). doi:10.1111/mpp.12569. 28520093 PMC6638078

[CIT0080] Zhao Z, Liu H, Wang C, Xu J-R. 2013. Comparative analysis of fungal genomes reveals different plant cell wall degrading capacity in fungi. *BMC Genomics*, 14, 274. doi:10.1186/1471-2164-14-274. PMC3652786 23617724

