# Supplementary material for: Genome-wide annotation, comparison and functional genomics of carbohydrate-active enzymes in legumes infecting Fusarium oxysporum formae speciales
Source: Mycology. 2020 Jan 4;11(1):56–70. doi: 10.1080/21501203.2019.1706656 (PMC7033727; doi:10.1080/21501203.2019.1706656)
Supplement: Supplemental Material [file TMYC_A_1706656_SM7838.docx]

**Supplementary tables:**

| S.no. | Organism | Strain | Host | Genome size | Total proteome | Resource |
| --- | --- | --- | --- | --- | --- | --- |
| 1. | *F.oxysporum* f. sp. *conglutinans* race2 | 54008 | Cabbage | 53.57 | 26246 | NCBI |
| 2. | *F.oxysporum* f. sp. *ciceris* | 38-1 | Chickpea | 54.18 | 16414 | Predicted |
| 3. | *F.oxysporum* f. sp. *lycopersici* | 4287 | Tomato | 61.38 | 27347 | NCBI |
| 4. | *F.oxysporum* f. sp. *radicis-cucmerinium* | Forc016 | Cucumber | 52.91 | 17168 | NCBI |
| 5. | *F.oxysporum* f. sp. *radicislycopersici* | 26381 | Tomato | 49.36 | 24739 | NCBI |
| 6. | *F.oxysporum* f. sp*. vasinfectum* | 25433 | Cotton | 52.91 | 25216 | NCBI |
| 7. | *F.oxysporum* f. sp. *medicaginis* | Fom-5190a | Alfalfa | 51.14 | 15741 | Predicted |
| 8. | *F.oxysporum* f. sp*. melonis* | 26406 | Melon | 54.03 | 26719 | NCBI |
| 9. | *F.oxysporum* f. sp. *pisi* | HDV247 | Pea | 55.19 | 26378 | NCBI |
| 10. | *F.oxysporum* f. sp. *raphani* | 54005 | Radish | 53.50 | 25666 | NCBI |
| 11. | *F.oxysporum* f. sp*. cepae* |  | Onion | 53.43 | 19342 | NCBI |
| 12. | *F.oxysporum* f. sp. *cubense* race 1 | Race 1 | Banana | 47.66 | 15438 | NCBI |

**Supplementary table S1.** Overview of the organisms belonging to the *formae speciales* of *Fusarium oxysporum* causing wilt disease

|  | **AA** | **CBM** | **CE** | **GH** | **GT** | **PL** | **GH+CBM** | **Total CAZymes** |
| --- | --- | --- | --- | --- | --- | --- | --- | --- |
| **FOC** | 80 | 3 | 44 | 301 | 105 | 23 | 17 | 573 |
| **FOMED** | 82 | 4 | 47 | 349 | 118 | 27 | 26 | 652 |
| **FOP** | 106 | 19 | 45 | 414 | 195 | 28 | 28 | 835 |
| **FOL** | 101 | 12 | 40 | 414 | 185 | 25 | 24 | 801 |
| **FOCEP** | 84 | 3 | 45 | 360 | 115 | 28 | 24 | 659 |
| **FOCU** | 77 | 3 | 41 | 328 | 112 | 26 | 20 | 607 |
| **FOCO** | 98 | 10 | 48 | 400 | 180 | 29 | 28 | 793 |
| **FOM** | 98 | 9 | 45 | 432 | 190 | 27 | 30 | 831 |
| **FORC** | 85 | 3 | 43 | 354 | 117 | 26 | 25 | 653 |
| **FORL** | 93 | 12 | 45 | 397 | 174 | 27 | 25 | 773 |
| **FOR** | 92 | 9 | 44 | 405 | 181 | 28 | 27 | 786 |
| **FOV** | 97 | 9 | 49 | 388 | 175 | 25 | 27 | 770 |

**Supplementary table S2**. Total numbers of CAZymes modules annotated using dbCAN2 among the *formae speciales* of *Fusarium oxysporum* causing wilt disease. **FOC**: *F. oxysporum f*. sp. *ciceris*, **FOMED**: *F. oxysporum* f. sp. *medicaginis*, **FOP**: *F. oxysporum* f. sp*. pisi*, **FOL**: *F. oxysporum* f. sp*. lycopersici*, **FOCEP***: F. oxysporum* f. sp*. cepae***, FOCU**: *F. oxysporum* f. sp. *cubense*, **FOCO**: *F. oxysporum* f. sp. *conglutinans*, **FOM**: *F. oxysporum* f. sp. *melonis*, **FORC**: *F. oxysporum* f. sp*. radicis-cucmerinum***, FORL***: F.oxysporum* f. sp*. radicis-lycopersici*, **FOR***: F. oxysporum* f. sp*. raphani*, **FOV**: *F. oxysporum* f.sp*. vasinfectum*,)

**AA:** Auxillary Activities, **CE**: Carbohydrate Esterase, **CBM**: Carbohydrate Binding Module, **GH**: Glycosyl hydrolase, **GT**: Glycosyl Transferase, **PL**: Polysaccharide lyase

|  | FOCO | FOC | FOL | FORC | FORL | FOV | FOMED | FOM | FOP | FOR | FOCU | FOCEP |
| --- | --- | --- | --- | --- | --- | --- | --- | --- | --- | --- | --- | --- |
| AA1 | 20 | 15 | 22 | 14 | 21 | 19 | 14 | 20 | 19 | 20 | 14 | 14 |
| AA11 | 4 | 4 | 4 | 4 | 4 | 4 | 4 | 4 | 4 | 4 | 4 | 4 |
| AA12 | 4 | 2 | 4 | 3 | 4 | 4 | 3 | 4 | 4 | 2 | 2 | 3 |
| AA13 | 1 | 1 | 1 | 1 | 1 | 1 | 1 | 2 | 1 | 1 | 1 | 1 |
| AA14 | 1 | 1 | 1 | 1 | 1 | 1 | 1 | 1 | 1 | 1 | 1 | 1 |
| AA2 | 2 | 1 | 2 | 1 | 2 | 2 | 2 | 2 | 2 | 2 | 1 | 1 |
| AA3 | 30 | 25 | 34 | 29 | 28 | 30 | 30 | 31 | 36 | 31 | 26 | 29 |
| AA4 | 0 | 3 | 2 | 2 | 1 | 0 | 0 | 1 | 2 | 0 | 0 | 2 |
| AA5 | 6 | 6 | 5 | 6 | 5 | 5 | 5 | 6 | 7 | 5 | 5 | 6 |
| AA6 | 2 | 2 | 1 | 1 | 1 | 1 | 1 | 1 | 1 | 1 | 1 | 1 |
| AA7 | 13 | 6 | 8 | 7 | 9 | 9 | 7 | 10 | 11 | 9 | 8 | 7 |
| AA8 | 1 | 2 | 1 | 1 | 1 | 1 | 0 | 1 | 1 | 1 | 1 | 1 |
| AA9 | 14 | 13 | 16 | 15 | 15 | 20 | 14 | 15 | 17 | 15 | 13 | 14 |
| CBM21 | 8 | 2 | 7 | 1 | 7 | 7 | 1 | 7 | 8 | 7 | 1 | 1 |
| CBM63 | 2 | 1 | 5 | 2 | 5 | 2 | 2 | 2 | 2 | 2 | 2 | 2 |
| CBM67 | 0 | 0 | 0 | 0 | 0 | 0 | 1 | 0 | 0 | 0 | 0 | 0 |
| GH10+CBM1 | 1 | 1 | 1 | 1 | 1 | 1 | 1 | 1 | 1 | 1 | 1 | 1 |
| GH15+CBM20 | 2 | 2 | 2 | 2 | 2 | 2 | 2 | 2 | 2 | 2 | 2 | 2 |
| GH18+CBM24 | 0 | 1 | 1 | 0 | 0 | 0 | 0 | 0 | 1 | 0 | 0 | 0 |
| GH32+CBM38 | 4 | 0 | 3 | 2 | 2 | 1 | 3 | 2 | 1 | 3 | 1 | 2 |
| GH43+CBM35 | 1 | 2 | 1 | 1 | 1 | 1 | 1 | 1 | 1 | 1 | 1 | 1 |
| GH43+CBM6 | 1 | 0 | 1 | 1 | 1 | 1 | 1 | 1 | 1 | 1 | 1 | 1 |
| GH54+CBM42 | 2 | 1 | 1 | 1 | 1 | 1 | 1 | 1 | 1 | 1 | 1 | 1 |
| GH7+CBM1 | 0 | 0 | 0 | 0 | 0 | 0 | 0 | 0 | 0 | 0 | 1 | 0 |
| GH71+CBM24 | 2 | 0 | 1 | 2 | 2 | 2 | 1 | 2 | 2 | 1 | 2 | 2 |
| GH72+CBM43 | 6 | 1 | 0 | 1 | 1 | 6 | 1 | 6 | 6 | 6 | 1 | 1 |
| GH78+CBM67 | 9 | 8 | 13 | 14 | 14 | 12 | 14 | 14 | 12 | 11 | 9 | 13 |
| GH78+GH79+CBM67 | 0 | 1 | 0 | 0 | 0 | 0 | 1 | 0 | 0 | 0 | 0 | 0 |
| CE1 | 4 | 2 | 3 | 4 | 4 | 5 | 4 | 5 | 5 | 3 | 3 | 4 |
| CE2 | 1 | 1 | 1 | 1 | 1 | 1 | 1 | 1 | 1 | 1 | 1 | 1 |
| CE3 | 6 | 9 | 5 | 7 | 6 | 7 | 8 | 5 | 5 | 5 | 7 | 6 |
| CE4 | 10 | 8 | 10 | 9 | 10 | 11 | 11 | 9 | 9 | 9 | 9 | 10 |
| CE5 | 10 | 10 | 7 | 8 | 9 | 9 | 10 | 9 | 9 | 9 | 8 | 8 |
| CE8 | 5 | 4 | 5 | 5 | 4 | 4 | 4 | 4 | 4 | 6 | 4 | 7 |
| CE9 | 4 | 1 | 1 | 1 | 3 | 4 | 1 | 3 | 3 | 3 | 1 | 1 |
| CE12 | 4 | 4 | 4 | 4 | 4 | 3 | 4 | 4 | 4 | 4 | 4 | 4 |
| CE16 | 4 | 4 | 4 | 4 | 4 | 5 | 4 | 4 | 4 | 4 | 4 | 4 |
| GH1 | 6 | 6 | 6 | 6 | 6 | 6 | 8 | 6 | 7 | 5 | 7 | 8 |
| GH2 | 9 | 9 | 8 | 10 | 11 | 7 | 10 | 10 | 9 | 10 | 8 | 11 |
| GH3 | 45 | 30 | 42 | 37 | 45 | 38 | 37 | 46 | 49 | 46 | 27 | 42 |
| GH5 | 21 | 19 | 21 | 23 | 21 | 23 | 22 | 22 | 23 | 21 | 23 | 23 |
| GH6 | 1 | 1 | 1 | 1 | 1 | 1 | 1 | 1 | 1 | 1 | 1 | 1 |
| GH7 | 6 | 3 | 3 | 3 | 6 | 6 | 3 | 3 | 6 | 6 | 1 | 3 |
| GH10 | 4 | 2 | 4 | 4 | 5 | 4 | 4 | 5 | 4 | 2 | 4 | 5 |
| GH11 | 4 | 3 | 4 | 3 | 3 | 4 | 3 | 4 | 4 | 4 | 3 | 3 |
| GH12 | 6 | 4 | 4 | 5 | 5 | 5 | 5 | 5 | 5 | 4 | 5 | 5 |
| GH13 | 14 | 11 | 16 | 10 | 15 | 14 | 11 | 18 | 17 | 14 | 8 | 9 |
| GH15 | 2 | 1 | 2 | 1 | 3 | 3 | 1 | 2 | 2 | 2 | 1 | 1 |
| GH16 | 36 | 23 | 35 | 27 | 35 | 34 | 26 | 36 | 36 | 40 | 28 | 26 |
| GH17 | 7 | 6 | 8 | 6 | 7 | 7 | 6 | 10 | 7 | 7 | 6 | 6 |
| GH18 | 29 | 27 | 33 | 33 | 20 | 29 | 23 | 34 | 26 | 30 | 24 | 26 |
| GH20 | 5 | 3 | 7 | 5 | 6 | 5 | 4 | 7 | 5 | 5 | 4 | 4 |
| GH24 | 2 | 1 | 1 | 2 | 2 | 2 | 2 | 3 | 2 | 2 | 2 | 2 |
| GH27 | 5 | 2 | 5 | 2 | 5 | 7 | 3 | 6 | 5 | 5 | 3 | 3 |
| GH28 | 14 | 11 | 17 | 13 | 12 | 14 | 12 | 13 | 12 | 18 | 12 | 13 |
| GH29 | 2 | 1 | 2 | 3 | 2 | 2 | 3 | 2 | 2 | 2 | 2 | 3 |
| GH30 | 1 | 1 | 3 | 2 | 2 | 1 | 2 | 3 | 2 | 2 | 2 | 2 |
| GH31 | 9 | 9 | 10 | 10 | 8 | 7 | 8 | 11 | 9 | 8 | 8 | 9 |
| GH32 | 10 | 5 | 13 | 8 | 8 | 7 | 9 | 11 | 8 | 10 | 9 | 8 |
| GH33 | 1 | 1 | 1 | 1 | 1 | 1 | 1 | 5 | 1 | 1 | 1 | 1 |
| GH35 | 5 | 2 | 7 | 5 | 7 | 5 | 7 | 8 | 8 | 4 | 5 | 8 |
| GH36 | 7 | 4 | 6 | 4 | 7 | 6 | 4 | 3 | 7 | 7 | 4 | 4 |
| GH37 | 4 | 2 | 2 | 2 | 4 | 4 | 2 | 5 | 5 | 5 | 2 | 2 |
| GH38 | 1 | 1 | 1 | 1 | 1 | 1 | 1 | 1 | 1 | 1 | 1 | 1 |
| GH39 | 4 | 4 | 4 | 4 | 4 | 4 | 3 | 4 | 4 | 4 | 4 | 4 |
| GH43 | 27 | 25 | 30 | 31 | 38 | 32 | 34 | 33 | 32 | 30 | 25 | 34 |
| GH45 | 3 | 1 | 2 | 1 | 2 | 2 | 1 | 2 | 2 | 2 | 1 | 1 |
| GH47 | 12 | 10 | 11 | 10 | 12 | 11 | 10 | 12 | 12 | 11 | 10 | 10 |
| GH49 | 1 | 1 | 1 | 1 | 1 | 1 | 1 | 1 | 1 | 1 | 1 | 1 |
| GH51 | 3 | 2 | 3 | 3 | 3 | 3 | 3 | 3 | 3 | 3 | 3 | 3 |
| GH53 | 1 | 1 | 2 | 1 | 1 | 2 | 1 | 2 | 2 | 1 | 2 | 1 |
| GH55 | 4 | 2 | 6 | 3 | 4 | 3 | 3 | 4 | 3 | 4 | 2 | 4 |
| GH62 | 1 | 1 | 1 | 1 | 1 | 1 | 1 | 1 | 1 | 1 | 1 | 1 |
| GH63 | 3 | 1 | 2 | 1 | 2 | 2 | 1 | 2 | 2 | 2 | 1 | 1 |
| GH64 | 6 | 4 | 9 | 3 | 4 | 4 | 2 | 4 | 6 | 4 | 7 | 3 |
| GH65 | 1 | 1 | 2 | 1 | 1 | 1 | 1 | 1 | 1 | 1 | 1 | 1 |
| GH67 | 2 | 1 | 2 | 3 | 3 | 1 | 3 | 3 | 3 | 3 | 2 | 3 |
| GH71 | 1 | 2 | 1 | 1 | 1 | 1 | 2 | 1 | 1 | 2 | 1 | 1 |
| GH72 | 2 | 2 | 3 | 2 | 2 | 2 | 2 | 2 | 2 | 2 | 2 | 2 |
| GH74 | 1 | 1 | 1 | 1 | 1 | 1 | 1 | 1 | 1 | 1 | 1 | 1 |
| GH75 | 3 | 2 | 2 | 2 | 2 | 2 | 2 | 3 | 3 | 3 | 2 | 2 |
| GH76 | 12 | 10 | 14 | 9 | 12 | 13 | 9 | 14 | 13 | 12 | 9 | 9 |
| GH78 | 5 | 3 | 5 | 5 | 5 | 6 | 4 | 4 | 5 | 5 | 6 | 4 |
| GH79 | 3 | 1 | 6 | 3 | 3 | 2 | 3 | 5 | 2 | 3 | 3 | 3 |
| GH81 | 3 | 1 | 3 | 1 | 3 | 3 | 1 | 3 | 3 | 3 | 1 | 1 |
| GH84 | 0 | 0 | 0 | 0 | 0 | 1 | 0 | 0 | 1 | 0 | 0 | 0 |
| GH88 | 3 | 3 | 3 | 3 | 3 | 2 | 3 | 3 | 3 | 3 | 3 | 3 |
| GH93 | 5 | 5 | 5 | 4 | 4 | 6 | 5 | 5 | 5 | 5 | 6 | 5 |
| GH95 | 2 | 1 | 2 | 2 | 2 | 2 | 2 | 2 | 2 | 1 | 2 | 2 |
| GH105 | 4 | 3 | 3 | 4 | 3 | 4 | 4 | 4 | 4 | 3 | 4 | 4 |
| GH106 | 2 | 2 | 1 | 2 | 2 | 2 | 4 | 3 | 3 | 4 | 3 | 2 |
| GH114 | 4 | 3 | 3 | 3 | 3 | 3 | 3 | 3 | 4 | 4 | 3 | 3 |
| GH115 | 2 | 3 | 1 | 3 | 4 | 4 | 3 | 3 | 4 | 4 | 3 | 3 |
| GH125 | 4 | 3 | 5 | 3 | 4 | 4 | 3 | 4 | 4 | 4 | 3 | 3 |
| GH127 | 2 | 2 | 2 | 2 | 2 | 2 | 2 | 2 | 2 | 2 | 1 | 2 |
| GH128 | 4 | 4 | 6 | 4 | 5 | 4 | 4 | 6 | 4 | 4 | 4 | 4 |
| GH131 | 1 | 1 | 0 | 1 | 1 | 1 | 1 | 1 | 1 | 1 | 1 | 1 |
| GH132 | 3 | 2 | 2 | 2 | 2 | 3 | 2 | 2 | 3 | 3 | 2 | 2 |
| GH134 | 3 | 0 | 2 | 1 | 2 | 3 | 1 | 2 | 3 | 1 | 2 | 1 |
| GH139 | 1 | 1 | 1 | 1 | 1 | 1 | 1 | 1 | 1 | 1 | 1 | 1 |
| GH142 | 1 | 0 | 1 | 1 | 1 | 1 | 1 | 1 | 0 | 0 | 1 | 1 |
| GH145 | 3 | 2 | 3 | 2 | 3 | 3 | 2 | 3 | 3 | 3 | 2 | 2 |
| GH146 | 1 | 1 | 1 | 1 | 1 | 1 | 1 | 1 | 1 | 1 | 1 | 1 |
| GH152 | 1 | 1 | 1 | 1 | 1 | 1 | 1 | 1 | 1 | 1 | 0 | 1 |
| GT1 | 16 | 12 | 20 | 16 | 19 | 15 | 20 | 22 | 21 | 18 | 16 | 17 |
| GT2 | 38 | 22 | 38 | 22 | 35 | 39 | 23 | 42 | 48 | 39 | 22 | 23 |
| GT3 | 2 | 1 | 3 | 1 | 2 | 2 | 1 | 2 | 2 | 3 | 1 | 1 |
| GT4 | 19 | 7 | 14 | 6 | 15 | 15 | 6 | 13 | 14 | 13 | 7 | 6 |
| GT8 | 11 | 7 | 14 | 8 | 10 | 11 | 8 | 10 | 10 | 12 | 7 | 8 |
| GT15 | 4 | 4 | 4 | 4 | 4 | 4 | 4 | 4 | 4 | 4 | 3 | 4 |
| GT17 | 2 | 2 | 2 | 2 | 2 | 3 | 2 | 3 | 3 | 2 | 2 | 2 |
| GT20 | 10 | 3 | 11 | 3 | 11 | 11 | 3 | 11 | 11 | 11 | 3 | 3 |
| GT21 | 1 | 1 | 1 | 1 | 1 | 1 | 1 | 1 | 1 | 1 | 1 | 1 |
| GT22 | 12 | 4 | 12 | 4 | 12 | 11 | 4 | 12 | 12 | 12 | 4 | 4 |
| GT24 | 1 | 1 | 1 | 1 | 1 | 1 | 1 | 1 | 1 | 1 | 1 | 1 |
| GT31 | 6 | 3 | 5 | 3 | 5 | 6 | 3 | 5 | 6 | 5 | 3 | 3 |
| GT32 | 6 | 5 | 6 | 7 | 6 | 6 | 6 | 6 | 7 | 7 | 6 | 6 |
| GT33 | 1 | 1 | 1 | 1 | 1 | 1 | 1 | 1 | 1 | 1 | 1 | 1 |
| GT34 | 6 | 3 | 6 | 3 | 6 | 6 | 3 | 7 | 6 | 6 | 3 | 3 |
| GT35 | 2 | 1 | 2 | 1 | 2 | 2 | 1 | 2 | 2 | 2 | 1 | 1 |
| GT39 | 4 | 3 | 4 | 3 | 4 | 4 | 3 | 4 | 4 | 4 | 3 | 3 |
| GT41 | 4 | 1 | 5 | 1 | 2 | 5 | 1 | 5 | 4 | 4 | 1 | 1 |
| GT48 | 1 | 1 | 1 | 1 | 1 | 1 | 1 | 1 | 1 | 1 | 1 | 1 |
| GT50 | 1 | 1 | 1 | 1 | 1 | 1 | 1 | 1 | 1 | 1 | 1 | 1 |
| GT54 | 1 | 0 | 1 | 1 | 1 | 1 | 1 | 1 | 1 | 1 | 1 | 1 |
| GT57 | 2 | 2 | 2 | 2 | 2 | 2 | 2 | 2 | 2 | 2 | 2 | 2 |
| GT58 | 1 | 1 | 1 | 1 | 1 | 1 | 1 | 1 | 1 | 1 | 1 | 1 |
| GT59 | 2 | 1 | 2 | 1 | 2 | 2 | 1 | 2 | 2 | 2 | 1 | 1 |
| GT62 | 6 | 3 | 6 | 3 | 7 | 5 | 3 | 8 | 8 | 8 | 3 | 3 |
| GT64 | 2 | 1 | 2 | 2 | 2 | 2 | 2 | 2 | 2 | 1 | 2 | 2 |
| GT66 | 1 | 1 | 1 | 1 | 1 | 1 | 1 | 1 | 1 | 1 | 1 | 1 |
| GT69 | 7 | 4 | 7 | 6 | 6 | 7 | 4 | 8 | 7 | 6 | 4 | 4 |
| GT71 | 3 | 3 | 3 | 3 | 3 | 3 | 3 | 3 | 3 | 3 | 3 | 3 |
| GT76 | 1 | 1 | 1 | 1 | 1 | 1 | 1 | 1 | 1 | 1 | 1 | 1 |
| GT90 | 7 | 5 | 8 | 7 | 8 | 5 | 6 | 8 | 8 | 8 | 6 | 6 |
| PL1 | 13 | 11 | 10 | 12 | 12 | 11 | 12 | 12 | 12 | 12 | 12 | 14 |
| PL3 | 7 | 6 | 7 | 7 | 7 | 7 | 7 | 7 | 7 | 7 | 7 | 7 |
| PL4 | 4 | 3 | 4 | 3 | 4 | 4 | 3 | 4 | 4 | 4 | 3 | 3 |
| PL9 | 2 | 2 | 2 | 2 | 2 | 2 | 2 | 2 | 2 | 2 | 2 | 2 |
| PL11 | 2 | 1 | 1 | 1 | 1 | 0 | 1 | 1 | 2 | 2 | 1 | 1 |
| PL26 | 1 | 0 | 1 | 1 | 1 | 1 | 1 | 1 | 1 | 1 | 1 | 1 |
|  | **793** | **573** | **801** | **653** | **773** | **770** | **652** | **830** | **825** | **786** | **607** | **659** |

**Supplementary table S3**: Family wise distribution of the CAZymes among the formae speciales of *Fusarium oxysporum* causing wilt disease

**(FOL**: *F. oxysporum* f. sp*. lycopersici*, **FORL***: F.oxysporum* f. sp*. radicis-lycopersici*, **FOCO**: *F. oxysporum* f. sp. *conglutinans*, **FOR***: F. oxysporum* f. sp*. raphani*, **FOV**: *F. oxysporum* f. sp*. vasinfectum*, **FOM**: *F. oxysporum* f. sp. *melonis*, **FOP**: *F. oxysporum* f. sp*. pisi*, **FOC**: *F. oxysporum f*. sp. *ciceris*, **FOCU**: *F. oxysporum* f. sp. *cubense*, **FORC**: *F. oxysporum* f. sp*. radicis-cucmerinum,* **FOMED**: *F. oxysporum* f. sp. *medicaginis*, **FOCEP***: F. oxysporum* f. sp*. cepae*). **AA:** Auxiliary Activities, **CE**: Carbohydrate Esterase, **CBM**: Carbohydrate Binding Module, **GH**: Glycosyl hydrolase, **GT**: Glycosyl Transferase, **PL**: Polysaccharide lyase

| CAZymes | Activity | FOC | FOMED | FOP |
| --- | --- | --- | --- | --- |
|  | **CELLULOSE DEGRADING** |  |  |  |
| GH1 | β-glucosidase | 6 | 8 | 7 |
| GH2 | β-galactosidase | 9 | 10 | 9 |
| GH3 | β-glucosidase | 30 | 37 | 49 |
| GH5 | endo-β-1,4-glucanase | 19 | 22 | 23 |
| GH6 | endoglucanase | 1 | 1 | 1 |
| GH7 | endo-β-1,4-glucanase | 3 | 3 | 6 |
| GH17 | glucan endo-1,3-β-glucosidase | 6 | 6 | 7 |
| GH31 | glucan endo-1,3-β-glucosidase | 9 | 8 | 9 |
| GH53 | endo-β-1,4-galactanase | 1 | 1 | 2 |
| GH55 | exo/endo-β-1,3-glucanase | 2 | 3 | 3 |
| GH71 | exo-β-1,3-glucanase | 2 | 2 | 1 |
| GH74 | endoglucanase | 1 | 1 | 1 |
| GH128 | β-1,3-glucanase | 4 | 4 | 4 |
| GH131 | exo-β-1,3/1,6-glucanase | 1 | 1 | 1 |
| GH132 | Activity on β-1,3-glucan | 2 | 2 | 3 |
| GH152 | β-1,3-glucanase | 1 | 1 | 1 |
| GH71+CBM24 | α-1,3-glucanase+α-1,3-glucan-binding function | 0 | 1 | 2 |
|  | **HEMICELLULOSE DEGRADING** |  |  |  |
| GH10 | endo-1,4/1,3-β-xylanase | 2 | 4 | 4 |
| GH11 | endo-1,4/1,3-β-xylanase | 3 | 3 | 4 |
| GH12 | xyloglucan hydrolase | 4 | 5 | 5 |
| GH16 | xyloglucosyltransferase | 23 | 26 | 36 |
| GH27 | α-galactosidase | 2 | 3 | 5 |
| GH30 | endo-β-1,4-xylanase | 1 | 2 | 2 |
| GH31 | α-glucosidase | 9 | 8 | 9 |
| GH35 | β-galactosidase | 2 | 7 | 8 |
| GH36 | α-galactosidase | 4 | 4 | 7 |
| GH39 | β-xylosidase | 4 | 3 | 4 |
| GH43 | β-xylosidase/xylanase | 25 | 34 | 32 |
| GH47 | α-mannosidase | 10 | 10 | 12 |
| GH51 | endo-β-1,4-xylanase | 2 | 3 | 3 |
| GH62 | α-L-arabinofuranosidase | 1 | 1 | 1 |
| GH95 | α-1,3-glucosidase | 1 | 2 | 2 |
| GH127 | α-L-fucosidase | 2 | 2 | 2 |
| GH146 | β-L-arabinofuranosidase | 1 | 1 | 1 |
| CE1 | β-L-arabinofuranosidase | 2 | 4 | 5 |
| CE2 | acetyl xylan esterase | 1 | 1 | 1 |
| CE3 | acetyl xylan esterase | 9 | 8 | 5 |
| CE4 | acetyl xylan esterase | 8 | 11 | 9 |
| CE5 | acetyl xylan esterase | 10 | 10 | 9 |
| CE12 | cutinase | 4 | 4 | 4 |
| CE16 | acetylesterase | 4 | 4 | 4 |
| GH10+CBM1 | endo-1,4/1,3-β-xylanase+cellulose-binding | 1 | 1 | 1 |
| GH43+CBM6 | xylanase+β-1,4-xylan binding | 0 | 1 | 1 |
|  | **PECTIN DEGRADING** |  |  |  |
| GH28 | polygalacturonase | 11 | 12 | 12 |
| GH38 | α-mannosidase | 1 | 1 | 1 |
| GH43 | β-xylosidase | 25 | 34 | 32 |
| GH53 | endo-β-1,4-galactanase | 1 | 1 | 2 |
| GH63 | α-glucosidase | 1 | 1 | 2 |
| GH76 | α-1,6-mannanase | 10 | 9 | 13 |
| GH78 | α-L-rhamnosidase | 3 | 4 | 5 |
| GH79 | β-glucuronidase | 1 | 3 | 2 |
| GH88 | β-glucuronyl hydrolase | 3 | 3 | 3 |
| GH93 | exo-α-L-1,5-arabinanase | 5 | 5 | 5 |
| GH105 | unsaturated rhamnogalacturonyl hydrolase | 3 | 4 | 4 |
| GH106 | α-L-rhamnosidase | 2 | 4 | 3 |
| GH125 | exo-α-1,6-mannosidase | 3 | 3 | 4 |
| GH145 | α-L-rhamnohydrolase | 2 | 2 | 3 |
| CE8 | pectin methylesterase | 4 | 4 | 4 |
| CE12 | rhamnogalacturonanacetylesterase | 4 | 4 | 4 |
| PL1 | pectatelyase | 11 | 12 | 12 |
| PL3 | pectatelyase | 6 | 7 | 7 |
| PL4 | rhamnogalacturonanendolyase | 3 | 3 | 4 |
| PL9 | exopolygalacturonatelyase | 2 | 2 | 2 |
| PL11 | rhamnogalacturonanexo/endolyase | 1 | 1 | 2 |
| PL20 | endo-β-1,4-glucuronan lyase | 0 | 1 | 0 |
| PL26 | rhamnogalacturonanexolyase | 0 | 1 | 1 |
| GH78+CBM67 | α-L-rhamnosidase+L-rhamnose binding | 8 | 14 | 12 |
|  | **LIGNIN DEGRADING AND LPMOs** |  |  |  |
| AA1 | Laccase | 15 | 14 | 19 |
| AA11 | lignin peroxidase | 4 | 4 | 4 |
| AA12 | cellobiose dehydrogenase | 2 | 3 | 4 |
| AA13 | vanillyl-alcohol oxidase | 1 | 1 | 1 |
| AA14 | galactose oxidase | 1 | 1 | 1 |
| AA2 | 1,4-benzoquinone reductase | 1 | 2 | 2 |
| AA3 | gluco/chito-oligosaccharide oxidase | 25 | 30 | 36 |
| AA4 | Iron reductase domain | 3 | 0 | 2 |
| AA5 | cellulose cleaving LPMO | 6 | 5 | 7 |
| AA6 | chitin cleaving LPMO | 2 | 1 | 1 |
| AA7 | quinone-dependent oxidoreductase | 6 | 7 | 11 |
| AA8 | starch cleaving LPMO | 2 | 0 | 1 |
| AA9 | xylan cleaving LPMO | 13 | 14 | 17 |

**Supplementary table S4**: Copy number distribution of the families involved in the process of plant cell wall degradation (PCWDE)
**AA:** Auxiliary Activities, **CE**: Carbohydrate Esterase, **CBM**: Carbohydrate Binding Module, **GH**: Glycosyl hydrolase, **PL**: Polysaccharide lyase

| Clusters | No. of clusters | Accessions | Uniprot id | Activity | Gene Ontology | e-value | HMMER | DIAMOND | HotPep |
| --- | --- | --- | --- | --- | --- | --- | --- | --- | --- |
| cluster1 | 7 | FOC\|g7018.t1;  FOC\|g13627.t1;  FOC\|g15426.t1;  FOC\|g5541.t1;  FOC\|g8349.t1;  FOMED\|g6568.t1;  FOP\|EXA33676.1; | Q01738 | Cellobiose dehydrogenase | GO:0005576; C:extracellular region; IEA:UniProtKB-KW; GO:0047735; F:cellobiose dehydrogenase (acceptor) activity; IEA:UniProtKB-EC; GO:0050660; F:flavin adenine dinucleotide binding; IEA:InterPro; GO:0046872; F:metal ion binding; IEA:UniProtKB-KW; GO:0030245; P:cellulose catabolic process; IEA:UniProtKB-KW | 7.80E-107 | AA3_1(7-745) | AA3 | AA3_1 |
| cluster55 | 3 | FOMED\|g3096.t1;  FOP\|EXA48985.1;  FOC\|g6911.t1; | P50325 | Chitin deacetylase | GO:0004099; F:chitin deacetylase activity; IEA:UniProtKB-EC; GO:0006032; P:chitin catabolic process; IEA:UniProtKB-KW; GO:0000272; P:polysaccharide catabolic process; IEA:UniProtKB-KW | 6.20E-19 | CE4(115-248) | CE4 | CBM18 |
| cluster131 | 3 | FOMED\|g8767.t1;  FOP\|EXA37835.1;  FOC\|g3761.t1; | P50325 | Chitin deacetylase | GO:0004099; F:chitin deacetylase activity; IEA:UniProtKB-EC; GO:0006032; P:chitin catabolic process; IEA:UniProtKB-KW; GO:0000272; P:polysaccharide catabolic process; IEA:UniProtKB-KW | 9.90E-25 | CE4(124-255) | CE4+CBM18 | CBM18 |
| cluster116 | 3 | FOMED\|g3632.t1;  FOP\|EXA47170.1;  FOC\|g12028.t1; | P49426 | Glucan 1,3-beta-glucosidase | GO:0004338; F:glucan exo-1,3-beta-glucosidase activity; IEA:UniProtKB-EC; GO:0071555; P:cell wall organization; IEA:UniProtKB-KW | 3.40E-160 | GH55(16-750) | GH55 | GH55 |
| cluster133 | 3 | FOMED\|g3025.t1;  FOP\|EXA49108.1;  FOC\|g5786.t1; | P49426 | Glucan 1,3-beta-glucosidase | GO:0004338; F:glucan exo-1,3-beta-glucosidase activity; IEA:UniProtKB-EC; GO:0071555; P:cell wall organization; IEA:UniProtKB-KW | 4.50E-166 | GH55(66-810) | GH55 | GH55 |
| cluster78 | 3 | FOMED\|g11887.t1  ;FOP\|EXA35092.1;  FOC\|g11027.t1; | O14405 | Endoglucanase-4 | GO:0005576; C:extracellular region; IDA:UniProtKB; GO:0008810; F:cellulase activity; IDA:UniProtKB; GO:0030248; F:cellulose binding; IEA:InterPro; GO:0030245; P:cellulose catabolic process; IDA:UniProtKB | 6.80E-60 | AA9(13-238) | AA9 | AA9 |
| cluster84 | 3 | FOMED\|g5430.t1;\  FOP\|EXA42154.1;  FOC\|g3435.t1; | O14405 | Endoglucanase-4 | GO:0005576; C:extracellular region; IDA:UniProtKB; GO:0008810; F:cellulase activity; IDA:UniProtKB; GO:0030248; F:cellulose binding; IEA:InterPro; GO:0030245; P:cellulose catabolic process; IDA:UniProtKB | 5.30E-87 | AA9(17-239) | AA9 | AA9 |
| cluster73 | 3 | FOMED\|g11869.t1;  FOP\|EXA35113.1;  FOC\|g10196.t1; | Q9HE18 | Feruloyl esterase B | GO:0005576; C:extracellular region; IDA:UniProtKB; GO:0030248; F:cellulose binding; IDA:UniProtKB; GO:0030600; F:feruloyl esterase activity; IDA:UniProtKB; GO:0004553; F:hydrolase activity, hydrolyzing O-glycosyl compounds; IEA:InterPro; GO:0016998; P:cell wall macromolecule catabolic process; IDA:UniProtKB; GO:0045490; P:pectin catabolic process; IDA:UniProtKB; GO:0045493; P:xylan catabolic process; IDA:UniProtKB | 8.60E-83 | CE1(36-201) | N | CE1 |
| cluster92 | 3 | FOMED\|g10128.t1;  FOP\|EXA36850.1;  FOC\|g9099.t1; | P11373 | Cutinase 1 | GO:0005576; C:extracellular region; IDA:UniProtKB; GO:0050525; F:cutinase activity; IDA:UniProtKB; GO:0009405; P:pathogenesis; IDA:UniProtKB | 5.50E-61 | CE5(53-228) | CE5 | N |
| cluster76 | 3 | FOMED\|g10362.t1;  FOP\|EXA36550.1;  FOC\|g6842.t1; | P46239 | Putative endoglucanase type F | GO:0005576; C:extracellular region; IEA:InterPro; GO:0008810; F:cellulase activity; IEA:UniProtKB-EC; GO:0030248; F:cellulose binding; IEA:InterPro; GO:0030245; P:cellulose catabolic process; IEA:UniProtKB-KW | 4.80E-227 | CBM1(22-49)+GH10(84-383) | GH10 | CBM1 |
| cluster9 | 4 | FOP\|EXA46150.1;  FOP\|EXA46149.1;  FOMED\|g8203.t1;  FOC\|g7944.t1; | P45699 | Putative endoglucanase type K | GO:0005576; C:extracellular region; IEA:InterPro; GO:0008810; F:cellulase activity; IEA:UniProtKB-EC; GO:0030248; F:cellulose binding; IEA:InterPro; GO:0030245; P:cellulose catabolic process; IEA:UniProtKB-KW | 1.20E-209 | GH45(68-266) | GH45 | CBM1 |
| cluster119 | 3 | FOMED\|g12448.t1;  FOP\|EXA34862.1;  FOC\|g8237.t1; | Q12624 | Endoglucanase 3 | GO:0005576; C:extracellular region; IEA:InterPro; GO:0008810; F:cellulase activity; IEA:UniProtKB-EC; GO:0030248; F:cellulose binding; IEA:InterPro; GO:0030245; P:cellulose catabolic process; IEA:UniProtKB-KW | 3.70E-131 | GH5_5(84-361) | GH5 | CBM1 |
| cluster56 | 3 | FOMED\|g9032.t1;  FOP\|EXA36251.1;  FOC\|g11867.t1; | P46236 | Putative endoglucanase type B | GO:0005576; C:extracellular region; IEA:InterPro; GO:0008810; F:cellulase activity; IEA:UniProtKB-EC; GO:0030248; F:cellulose binding; IEA:InterPro; GO:0030245; P:cellulose catabolic process; IEA:UniProtKB-KW | 5.70E-275 | GH6(130-422) | GH6 | CBM1 |
| cluster2 | 6 | FOP\|EXA52997.1;  FOP\|EXA52996.1;  FOP\|EXA52998.1;  FOP\|EXA52999.1;  FOMED\|g857.t1;  FOC\|g743.t1; | P46238 | Putative exoglucanase type C | GO:0005576; C:extracellular region; IEA:InterPro; GO:0016162; F:cellulose 1,4-beta-cellobiosidase activity; IEA:UniProtKB-EC; GO:0030248; F:cellulose binding; IEA:InterPro; GO:0030245; P:cellulose catabolic process; IEA:UniProtKB-KW | 0 | GH7(19-451) | GH7 | CBM1 |
| cluster60 | 3 | FOMED\|g10702.t1;  FOP\|EXA35575.1;  FOC\|g1993.t1; | P46238 | Putative exoglucanase type C | GO:0005576; C:extracellular region; IEA:InterPro; GO:0016162; F:cellulose 1,4-beta-cellobiosidase activity; IEA:UniProtKB-EC; GO:0030248; F:cellulose binding; IEA:InterPro; GO:0030245; P:cellulose catabolic process; IEA:UniProtKB-KW | 5.50E-60 | GH7(23-429) | N | GH7 |
| cluster23 | 3 | FOMED\|g10127.t1  ;FOP\|EXA36851.1  ;FOC\|g9098.t1; | Q7Z9M8 | Xyloglucanase {ECO:0000303\|PubMed:15541296} | GO:0005576; C:extracellular region; IEA:InterPro; GO:0030248; F:cellulose binding; IEA:InterPro; GO:0033950; F:xyloglucan-specific exo-beta-1,4-glucanase activity; IEA:UniProtKB-EC; GO:0030245; P:cellulose catabolic process; IEA:UniProtKB-KW | 2.90E-297 | GH74(82-179) | GH74 | GH74 |
| cluster30 | 3 | FOMED\|g8524.t1;  FOP\|EXA33519.1;  FOC\|g8629.t1; | Q700S9 | Probable beta-galactosidase A | GO:0005576; C:extracellular region; IEA:UniProtKB-KW; GO:0004565; F:beta-galactosidase activity; IEA:UniProtKB-EC; GO:0000272; P:polysaccharide catabolic process; IEA:UniProtKB-KW | 0 | GH35(48-378) | GH35 | GH35 |
| cluster8 | 4 | FOP\|EXA46299.1;  FOP\|EXA46298.1;  FOMED\|g8309.t1;  FOC\|g12586.t1; | Q873X9 | Endochitinase B1 | GO:0005576; C:extracellular region; IEA:UniProtKB-KW; GO:0004568; F:chitinase activity; IEA:UniProtKB-EC; GO:0006032; P:chitin catabolic process; IEA:UniProtKB-KW; GO:0000272; P:polysaccharide catabolic process; IEA:UniProtKB-KW | 3.00E-20 | GH18(21-423) | GH18 | GH18 |
| cluster110 | 3 | FOMED\|g3193.t1;  FOP\|EXA48855.1;  FOC\|g11400.t1; | Q1ZZM3 | Probable rhamnogalacturonase E | GO:0005576; C:extracellular region; IEA:UniProtKB-KW; GO:0004650; F:polygalacturonase activity; IEA:InterPro; GO:0071555; P:cell wall organization; IEA:UniProtKB-KW; GO:0000272; P:polysaccharide catabolic process; IEA:UniProtKB-KW | 1.10E-79 | GH28(149-396) | GH28 | GH28 |
| cluster121 | 3 | FOMED\|g9276.t1;  FOP\|EXA35935.1;  FOC\|g11884.t1; | Q9UUZ2 | Endo-xylogalacturonan hydrolase A | GO:0005576; C:extracellular region; IEA:UniProtKB-KW; GO:0004650; F:polygalacturonase activity; IEA:InterPro; GO:0071555; P:cell wall organization; IEA:UniProtKB-KW; GO:0000272; P:polysaccharide catabolic process; IEA:UniProtKB-KW | 6.40E-105 | GH28(59-397) | GH28 | GH28 |
| cluster54 | 3 | FOMED\|g11880.t1;  FOP\|EXA35101.1;  FOC\|g11021.t1; | Q07181 | Polygalacturonase | GO:0005576; C:extracellular region; IEA:UniProtKB-KW; GO:0004650; F:polygalacturonase activity; IEA:UniProtKB-EC; GO:0005975; P:carbohydrate metabolic process; IEA:InterPro; GO:0071555; P:cell wall organization; IEA:UniProtKB-KW | 1.40E-191 | GH28(37-362) | GH28 | GH28 |
| cluster95 | 3 | FOMED\|g11043.t1;  FOP\|EXA32498.1;  FOC\|g16249.t1; | Q00446 | Endopolygalacturonase 1 | GO:0005576; C:extracellular region; IEA:UniProtKB-KW; GO:0004650; F:polygalacturonase activity; IEA:UniProtKB-EC; GO:0005975; P:carbohydrate metabolic process; IEA:InterPro; GO:0071555; P:cell wall organization; IEA:UniProtKB-KW | 1.20E-152 | GH28(39-353) | GH28 | GH28 |
| cluster98 | 3 | FOMED\|g4712.t1;  FOP\|EXA44373.1;  FOC\|g3511.t1; | P17489 | Laccase-1 | GO:0005576; C:extracellular region; IEA:UniProtKB-KW; GO:0005507; F:copper ion binding; IEA:InterPro; GO:0052716; F:hydroquinone:oxygen oxidoreductase activity; IDA:ASPGD; GO:0048315; P:conidium formation; IMP:ASPGD; GO:0043324; P:pigment metabolic process involved in developmental pigmentation; IMP:ASPGD; GO:0030435; P:sporulation resulting in formation of a cellular spore; IEA:UniProtKB-KW | 7.10E-100 | N | AA1 | AA1 |
| cluster101 | 3 | FOMED\|g10369.t1;  FOP\|EXA36542.1;  FOC\|g6849.t1; | Q96WM9 | Laccase-2 | GO:0005576; C:extracellular region; IEA:UniProtKB-KW; GO:0005507; F:copper ion binding; IEA:InterPro; GO:0052716; F:hydroquinone:oxygen oxidoreductase activity; IEA:UniProtKB-EC; GO:0046274; P:lignin catabolic process; IEA:UniProtKB-KW | 8.50E-156 | AA1_3(48-363) | AA1 | AA1_3 |
| cluster103 | 3 | FOMED\|g3441.t1;  FOP\|EXA47524.1  FOC\|g2318.t1; | Q96WM9 | Laccase-2 | GO:0005576; C:extracellular region; IEA:UniProtKB-KW; GO:0005507; F:copper ion binding; IEA:InterPro; GO:0052716; F:hydroquinone:oxygen oxidoreductase activity; IEA:UniProtKB-EC; GO:0046274; P:lignin catabolic process; IEA:UniProtKB-KW | 1.20E-138 | AA1_3(51-365) | AA1 | AA1_3 |
| cluster6 | 5 | FOMED\|g11078.t1;  FOP\|EXA41560.1;  FOC\|g12131.t1;  FOC\|g6737.t1;  FOP\|EXA30682.1; | B8N5S6 | Probable beta-glucosidase M | GO:0005576; C:extracellular region; IEA:UniProtKB-KW; GO:0008422; F:beta-glucosidase activity; IEA:UniProtKB-EC; GO:0030245; P:cellulose catabolic process; IEA:UniProtKB-UniPathway | 2.90E-237 | GH3(134-347) | GH3 | GH3 |
| cluster66 | 3 | FOMED\|g5149.t1;  FOP\|EXA41696.1;  FOC\|g8523.t1; | A1D4F1 | Probable xyloglucan-specific endo-beta-1,4-glucanase A | GO:0005576; C:extracellular region; IEA:UniProtKB-KW; GO:0008810; F:cellulase activity; IEA:InterPro; GO:0033946; F:xyloglucan-specific endo-beta-1,4-glucanase activity; IEA:UniProtKB-EC; GO:0071555; P:cell wall organization; IEA:UniProtKB-KW; GO:0000272; P:polysaccharide catabolic process; IEA:UniProtKB-KW | 9.40E-67 | GH12(100-248) | GH12 | GH12 |
| cluster75 | 3 | FOMED\|g9036.t1;  FOP\|EXA36247.1;  FOC\|g11871.t1; | A1DBS6 | Probable endo-beta-1,4-glucanase D | GO:0005576; C:extracellular region; IEA:UniProtKB-KW; GO:0008810; F:cellulase activity; IEA:UniProtKB-EC; GO:0030248; F:cellulose binding; IEA:InterPro; GO:0030245; P:cellulose catabolic process; IEA:UniProtKB-KW | 3.80E-31 | AA9(6-230) | AA9 | AA9 |
| cluster115 | 3 | FOMED\|g12483.t1;  FOP\|EXA34813.1;  FOC\|g8274.t1; | B0Y9G4 | Probable endo-beta-1,4-glucanase D | GO:0005576; C:extracellular region; IEA:UniProtKB-KW; GO:0008810; F:cellulase activity; IEA:UniProtKB-EC; GO:0030248; F:cellulose binding; IEA:InterPro; GO:0030245; P:cellulose catabolic process; IEA:UniProtKB-KW | 2.10E-36 | AA9(7-210) | AA9 | AA9 |
| cluster125 | 3 | FOMED\|g12535.t1;  FOP\|EXA34126.1;  FOC\|g10429.t1; | O13990 | Glucan 1,3-beta-glucosidase | GO:0005576; C:extracellular region; IEA:UniProtKB-KW; GO:0009277; C:fungal-type cell wall; ISO:PomBase; GO:0004338; F:glucan exo-1,3-beta-glucosidase activity; ISO:PomBase; GO:0070879; P:fungal-type cell wall beta-glucan metabolic process; IC:PomBase | 5.50E-53 | GH17(49-300) | GH17 | GH17 |
| cluster107 | 3 | FOMED\|g1595.t1;  FOP\|EXA41318.1;  FOC\|g894.t1; | Q4WB37 | Endo-chitosanase | GO:0005576; C:extracellular region; IEA:UniProtKB-KW; GO:0016977; F:chitosanase activity; IDA:ASPGD; GO:0000272; P:polysaccharide catabolic process; IEA:UniProtKB-KW | 1.90E-82 | GH75(7-225) | GH75 | GH75 |
| cluster47 | 3 | FOMED\|g4171.t1;  FOP\|EXA29056.1;  FOC\|g7111.t1; | Q5B833 | Mannan endo-1,4-beta-mannosidase B | GO:0005576; C:extracellular region; IEA:UniProtKB-KW; GO:0016985; F:mannan endo-1,4-beta-mannosidase activity; IDA:UniProtKB; GO:0046355; P:mannan catabolic process; IDA:UniProtKB | 4.80E-102 | GH5_7(52-331) | N | GH5_7 |
| cluster85 | 3 | FOMED\|g923.t1;  FOP\|EXA53133.1;  FOC\|g809.t1; | Q4W9T6 | Probable rhamnogalacturonate lyase A | GO:0005576; C:extracellular region; IEA:UniProtKB-KW; GO:0030246; F:carbohydrate binding; IEA:InterPro; GO:0016837; F:carbon-oxygen lyase activity, acting on polysaccharides; IEA:InterPro; GO:0071555; P:cell wall organization; IEA:UniProtKB-KW; GO:0000272; P:polysaccharide catabolic process; IEA:UniProtKB-KW | 2.70E-183 | PL4_1(16-529) | PL4 | PL4_1 |
| cluster34 | 3 | FOMED\|g8046.t1;  FOP\|EXA45845.1;  FOC\|g3964.t1; | G2Q9T3 | Polysaccharide monooxygenase Cel61a | GO:0005576; C:extracellular region; IEA:UniProtKB-KW; GO:0030248; F:cellulose binding; IEA:InterPro; GO:0004553; F:hydrolase activity, hydrolyzing O-glycosyl compounds; IEA:InterPro; GO:0016491; F:oxidoreductase activity; IEA:UniProtKB-KW; GO:0030245; P:cellulose catabolic process; IEA:UniProtKB-KW | 2.00E-40 | AA9(6-233) | AA9 | AA9 |
| cluster126 | 3 | FOMED\|g9080.t1;  FOP\|EXA36188.1;  FOC\|g12053.t1; | G2Q9T3 | Polysaccharide monooxygenase Cel61a | GO:0005576; C:extracellular region; IEA:UniProtKB-KW; GO:0030248; F:cellulose binding; IEA:InterPro; GO:0004553; F:hydrolase activity, hydrolyzing O-glycosyl compounds; IEA:InterPro; GO:0016491; F:oxidoreductase activity; IEA:UniProtKB-KW; GO:0030245; P:cellulose catabolic process; IEA:UniProtKB-KW | 1.40E-59 | AA9(9-227) | AA9 | AA9 |
| cluster108 | 3 | FOMED\|g4726.t1;  FOP\|EXA44354.1;  FOC\|g3496.t1; | P79046 | Endo-1,4-beta-xylanase 1 | GO:0005576; C:extracellular region; IEA:UniProtKB-KW; GO:0030248; F:cellulose binding; IEA:InterPro; GO:0031176; F:endo-1,4-beta-xylanase activity; IEA:UniProtKB-EC; GO:0045493; P:xylan catabolic process; IEA:UniProtKB-UniPathway | 1.20E-122 | GH10(20-314) | GH10+CBM1+CBM22 | CBM1 |
| cluster93 | 3 | FOMED\|g8520.t1;  FOP\|EXA33524.1;  FOC\|g8634.t1; | Q5ATC7 | Pectate lyase H | GO:0005576; C:extracellular region; IEA:UniProtKB-KW; GO:0030570; F:pectate lyase activity; IDA:UniProtKB; GO:0071555; P:cell wall organization; IEA:UniProtKB-KW; GO:0045490; P:pectin catabolic process; IDA:UniProtKB | 2.80E-44 | PL3_2(58-245) | N | PL3_2 |
| cluster74 | 3 | FOMED\|g4076.t1;  FOP\|EXA46476.1;  FOC\|g8357.t1; | Q5B024 | Probable pectate lyase F | GO:0005576; C:extracellular region; IEA:UniProtKB-KW; GO:0030570; F:pectate lyase activity; IEA:UniProtKB-EC; GO:0071555; P:cell wall organization; IEA:UniProtKB-KW; GO:0000272; P:polysaccharide catabolic process; IEA:UniProtKB-KW | 1.30E-75 | PL3_2(29-215) | PL3 | PL3_2 |
| cluster111 | 3 | FOMED\|g10051.t1;  FOP\|EXA32330.1;  FOC\|g9903.t1; | Q5B024 | Probable pectate lyase F | GO:0005576; C:extracellular region; IEA:UniProtKB-KW; GO:0030570; F:pectate lyase activity; IEA:UniProtKB-EC; GO:0071555; P:cell wall organization; IEA:UniProtKB-KW; GO:0000272; P:polysaccharide catabolic process; IEA:UniProtKB-KW | 8.50E-78 | PL3_2(29-217) | PL3 | PL3_2 |
| cluster97 | 3 | FOMED\|g8280.t1;  FOP\|EXA46269.1  ;FOC\|g12240.t1; | A1C4B8 | Probable pectate lyase E | GO:0005576; C:extracellular region; IEA:UniProtKB-KW; GO:0030570; F:pectate lyase activity; IEA:UniProtKB-EC; GO:0071555; P:cell wall organization; IEA:UniProtKB-KW; GO:0000272; P:polysaccharide catabolic process; IEA:UniProtKB-KW | 4.00E-89 | PL3_2(42-228) | PL3 | PL3_2 |
| cluster114 | 3 | FOMED\|g9132.t1;  FOP\|EXA36112.1;  FOC\|g12768.t1; | Q0CJ49 | Probable pectate lyase D | GO:0005576; C:extracellular region; IEA:UniProtKB-KW; GO:0030570; F:pectate lyase activity; IEA:UniProtKB-EC; GO:0071555; P:cell wall organization; IEA:UniProtKB-KW; GO:0000272; P:polysaccharide catabolic process; IEA:UniProtKB-KW | 1.70E-59 | PL3_2(44-233) | PL3 | PL3_2 |
| cluster36 | 3 | FOMED\|g12747.t1;  FOP\|EXA34191.1;  FOC\|g8011.t1; | I1S2K3 | Endo-1,4-beta-xylanase A | GO:0005576; C:extracellular region; IEA:UniProtKB-KW; GO:0031176; F:endo-1,4-beta-xylanase activity; IEA:UniProtKB-EC; GO:0009405; P:pathogenesis; IEA:UniProtKB-KW; GO:0045493; P:xylan catabolic process; IEA:UniProtKB-UniPathway | 6.80E-128 | GH11(51-226) | GH11 | GH11 |
| cluster104 | 3 | FOMED\|g9027.t1;  FOP\|EXA36256.1;  FOC\|g11862.t1; | I1RII8 | Endo-1,4-beta-xylanase B | GO:0005576; C:extracellular region; IEA:UniProtKB-KW; GO:0031176; F:endo-1,4-beta-xylanase activity; IEA:UniProtKB-EC; GO:0009405; P:pathogenesis; IEA:UniProtKB-KW; GO:0045493; P:xylan catabolic process; IEA:UniProtKB-UniPathway | 2.60E-103 | GH11(54-228) | GH11 | GH11 |
| cluster134 | 3 | FOMED\|g6226.t1;  FOP\|EXA43437.1;  FOC\|g10762.t1; | Q92245 | Endo-1,4-beta-xylanase 4 | GO:0005576; C:extracellular region; IEA:UniProtKB-KW; GO:0031176; F:endo-1,4-beta-xylanase activity; IEA:UniProtKB-EC; GO:0045493; P:xylan catabolic process; IEA:UniProtKB-UniPathway | 8.10E-86 | GH11(44-219) | GH11 | GH11 |
| cluster31 | 3 | FOMED\|g12116.t1;  FOP\|EXA51142.1;  FOC\|g13296.t1; | P48845 | Dextranase | GO:0005576; C:extracellular region; IEA:UniProtKB-KW; GO:0033904; F:dextranase activity; IEA:UniProtKB-EC | 5.10E-159 | GH49(24-598) | GH49 | GH49 |
| cluster3 | 5 | FOP\|EXA29298.1;  FOP\|EXA31607.1;  FOP\|EXA33241.1;  FOMED\|g8960.t1;  FOC\|g10101.t1; | I1S2N3 | Galactose oxidase | GO:0005576; C:extracellular region; IEA:UniProtKB-KW; GO:0045480; F:galactose oxidase activity; IEA:UniProtKB-EC; GO:0046872; F:metal ion binding; IEA:UniProtKB-KW; GO:0007155; P:cell adhesion; IEA:InterPro | 6.60E-248 | AA5_2(54-677) | AA5 | AA5_2 |
| cluster88 | 3 | FOMED\|g14027.t1;  FOP\|EXA32528.1;  FOC\|g9703.t1; | I1S2N3 | Galactose oxidase | GO:0005576; C:extracellular region; IEA:UniProtKB-KW; GO:0045480; F:galactose oxidase activity; IEA:UniProtKB-EC; GO:0046872; F:metal ion binding; IEA:UniProtKB-KW; GO:0007155; P:cell adhesion; IEA:InterPro | 3.80E-128 | AA5_2(96-708) | AA5 | AA5_2 |
| cluster61 | 3 | FOMED\|g11145.t1;  FOP\|EXA39657.1  ;FOC\|g10478.t1; | B8NBI2 | Probable acetylxylan esterase A | GO:0005576; C:extracellular region; IEA:UniProtKB-KW; GO:0046555; F:acetylxylan esterase activity; IEA:UniProtKB-EC; GO:0030245; P:cellulose catabolic process; IEA:UniProtKB-KW; GO:0045493; P:xylan catabolic process; IEA:UniProtKB-UniPathway | 5.60E-98 | CE1(42-233) | CE1 | CE1 |
| cluster67 | 3 | FOMED\|g4882.t1;  FOP\|EXA44112.1;  FOC\|g11515.t1; | Q99034 | Acetylxylan esterase | GO:0005576; C:extracellular region; IEA:UniProtKB-KW; GO:0046555; F:acetylxylan esterase activity; IEA:UniProtKB-EC; GO:0030248; F:cellulose binding; IEA:InterPro; GO:0004553; F:hydrolase activity, hydrolyzing O-glycosyl compounds; IEA:InterPro; GO:0030245; P:cellulose catabolic process; IEA:UniProtKB-KW; GO:0045493; P:xylan catabolic process; IEA:UniProtKB-UniPathway | 7.30E-74 | CE5(22-227) | CE5 | CE5 |
| cluster62 | 3 | FOMED\|g255.t1;  FOP\|EXA51724.1;  FOC\|g130.t1; | Q99034 | Acetylxylan esterase | GO:0005576; C:extracellular region; IEA:UniProtKB-KW; GO:0046555; F:acetylxylan esterase activity; IEA:UniProtKB-EC; GO:0030248; F:cellulose binding; IEA:InterPro; GO:0004553; F:hydrolase activity, hydrolyzing O-glycosyl compounds; IEA:InterPro; GO:0030245; P:cellulose catabolic process; IEA:UniProtKB-KW; GO:0045493; P:xylan catabolic process; IEA:UniProtKB-UniPathway | 3.90E-27 | CE5(28-213) | N | CE5 |
| cluster81 | 3 | FOMED\|g12125.t1;  FOP\|EXA51152.1;  FOC\|g13305.t1; | Q2U7D2 | Probable alpha-L-arabinofuranosidase axhA | GO:0005576; C:extracellular region; IEA:UniProtKB-KW; GO:0046556; F:alpha-L-arabinofuranosidase activity; IEA:UniProtKB-EC; GO:0046373; P:L-arabinose metabolic process; IEA:InterPro; GO:0045493; P:xylan catabolic process; IEA:UniProtKB-KW | 4.60E-130 | GH62(28-296) | GH62 | GH62 |
| cluster132 | 3 | FOMED\|g9414.t1  ;FOP\|EXA35729.1;  FOC\|g8131.t1; | Q5B6Q3 | Glucan endo-1,6-beta-glucosidase B | GO:0005576; C:extracellular region; IEA:UniProtKB-KW; GO:0046557; F:glucan endo-1,6-beta-glucosidase activity; IDA:ASPGD; GO:0071555; P:cell wall organization; IEA:UniProtKB-KW; GO:0009251; P:glucan catabolic process; IDA:ASPGD | 3.90E-108 | GH5_15(82-384) | GH5 | GH5_15 |
| cluster39 | 3 | FOMED\|g13372.t1;  FOP\|EXA33117.1  ;FOC\|g13360.t1; | Q5AZC8 | Arabinan endo-1,5-alpha-L-arabinosidase B | GO:0005576; C:extracellular region; IEA:UniProtKB-KW; GO:0046558; F:arabinan endo-1,5-alpha-L-arabinosidase activity; IDA:UniProtKB; GO:0031222; P:arabinan catabolic process; IEA:UniProtKB-UniPathway; GO:0045490; P:pectin catabolic process; IDA:UniProtKB; GO:0045493; P:xylan catabolic process; IEA:UniProtKB-KW | 7.30E-09 | GH43_29(24-302)+CBM6(325-449) | GH43 | CBM6 |
| cluster44 | 3 | FOMED\|g6439.t1;  FOP\|EXA43833.1;  FOC\|g9517.t1; | A5AAG2 | Probable arabinan endo-1,5-alpha-L-arabinosidase C | GO:0005576; C:extracellular region; IEA:UniProtKB-KW; GO:0046558; F:arabinan endo-1,5-alpha-L-arabinosidase activity; IEA:UniProtKB-EC; GO:0031222; P:arabinan catabolic process; IEA:UniProtKB-UniPathway; GO:0045493; P:xylan catabolic process; IEA:UniProtKB-KW | 0.00011 | GH43_26(21-316) | N | GH43_26 |
| cluster29 | 3 | FOMED\|g10901.t1;  FOP\|EXA34513.1  ;FOC\|g9240.t1; | A1D5W1 | Probable arabinan endo-1,5-alpha-L-arabinosidase A | GO:0005576; C:extracellular region; IEA:UniProtKB-KW; GO:0046558; F:arabinan endo-1,5-alpha-L-arabinosidase activity; IEA:UniProtKB-EC; GO:0031222; P:arabinan catabolic process; IEA:UniProtKB-UniPathway; GO:0045493; P:xylan catabolic process; IEA:UniProtKB-KW | 2.30E-57 | GH43_5(34-312) | GH43 | GH43_5 |
| cluster38 | 3 | FOMED\|g9033.t1;  FOP\|EXA36250.1;  FOC\|g11868.t1; | Q99024 | Alpha-glucuronidase | GO:0005576; C:extracellular region; IEA:UniProtKB-KW; GO:0046559; F:alpha-glucuronidase activity; IEA:UniProtKB-EC; GO:0045493; P:xylan catabolic process; IEA:UniProtKB-KW | 0 | GH67(18-697) | GH67 | GH67 |
| cluster89 | 3 | FOMED\|g10190.t1;  FOP\|EXA36773.1;  FOC\|g14636.t1; | Q00645 | Pectate lyase plyB | GO:0005576; C:extracellular region; IEA:UniProtKB-KW; GO:0046872; F:metal ion binding; IEA:UniProtKB-KW; GO:0030570; F:pectate lyase activity; IDA:UniProtKB; GO:0071555; P:cell wall organization; IEA:UniProtKB-KW; GO:0045490; P:pectin catabolic process; IDA:UniProtKB | 3.40E-101 | PL1_7(72-253) | PL1 | PL1_7 |
| cluster112 | 3 | FOMED\|g8521.t1;  FOP\|EXA33523.1;  FOC\|g8633.t1; | Q00645 | Pectate lyase plyB | GO:0005576; C:extracellular region; IEA:UniProtKB-KW; GO:0046872; F:metal ion binding; IEA:UniProtKB-KW; GO:0030570; F:pectate lyase activity; IDA:UniProtKB; GO:0071555; P:cell wall organization; IEA:UniProtKB-KW; GO:0045490; P:pectin catabolic process; IDA:UniProtKB | 8.90E-134 | PL1_7(76-257) | PL1 | PL1_7 |
| cluster68 | 3 | FOMED\|g3803.t1;  FOP\|EXA46875.1;  FOC\|g2848.t1; | O59939 | Pectate lyase B | GO:0005576; C:extracellular region; IEA:UniProtKB-KW; GO:0046872; F:metal ion binding; IEA:UniProtKB-KW; GO:0030570; F:pectate lyase activity; IEA:UniProtKB-EC; GO:0009405; P:pathogenesis; IEA:UniProtKB-KW; GO:0045490; P:pectin catabolic process; IEA:UniProtKB-UniPathway | 2.50E-107 | PL1_7(79-260) | PL1 | PL1_7 |
| cluster128 | 3 | FOMED\|g3718.t1;  FOP\|EXA47010.1;  FOC\|g2933.t1; | B0XMA2 | Probable pectate lyase C | GO:0005576; C:extracellular region; IEA:UniProtKB-KW; GO:0046872; F:metal ion binding; IEA:UniProtKB-KW; GO:0030570; F:pectate lyase activity; IEA:UniProtKB-EC; GO:0071555; P:cell wall organization; IEA:UniProtKB-KW; GO:0000272; P:polysaccharide catabolic process; IEA:UniProtKB-KW | 1.20E-154 | PL1_2(88-263) | PL1 | PL1_2 |
| cluster96 | 3 | FOMED\|g13033.t1;  FOP\|EXA32612.1;  FOC\|g13275.t1; | Q00374 | Pectin lyase | GO:0005576; C:extracellular region; IEA:UniProtKB-KW; GO:0047490; F:pectin lyase activity; IEA:UniProtKB-EC | 2.70E-126 | PL1_4(111-296) | PL1 | PL1_4 |
| cluster59 | 3 | FOMED\|g12589.t1;  FOP\|EXA34058.1;  FOC\|g12827.t1; | Q00374 | Pectin lyase | GO:0005576; C:extracellular region; IEA:UniProtKB-KW; GO:0047490; F:pectin lyase activity; IEA:UniProtKB-EC | 1.50E-132 | PL1_4(113-297) | PL1 | PL1_4 |
| cluster64 | 3 | FOMED\|g10271.t1  ;FOP\|EXA36678.1;  FOC\|g7134.t1; | Q0CZD4 | Probable pectin lyase F-2 | GO:0005576; C:extracellular region; IEA:UniProtKB-KW; GO:0047490; F:pectin lyase activity; ISS:UniProtKB; GO:0071555; P:cell wall organization; IEA:UniProtKB-KW; GO:0045490; P:pectin catabolic process; ISS:UniProtKB | 1.60E-90 | PL1_4(108-294) | N | CBM1 |
| cluster100 | 3 | FOMED\|g3197.t1  ;FOP\|EXA48850.1;  FOC\|g11396.t1; | Q2UCT7 | Probable pectin lyase C | GO:0005576; C:extracellular region; IEA:UniProtKB-KW; GO:0047490; F:pectin lyase activity; ISS:UniProtKB; GO:0071555; P:cell wall organization; IEA:UniProtKB-KW; GO:0045490; P:pectin catabolic process; ISS:UniProtKB | 9.10E-114 | PL1_4(109-293) | PL1 | PL1_4 |
| cluster79 | 3 | FOMED\|g9739.t1;  FOP\|EXA54450.1;  FOC\|g2517.t1; | Q4WV10 | Probable pectin lyase A | GO:0005576; C:extracellular region; IEA:UniProtKB-KW; GO:0047490; F:pectin lyase activity; ISS:UniProtKB; GO:0071555; P:cell wall organization; IEA:UniProtKB-KW; GO:0045490; P:pectin catabolic process; ISS:UniProtKB | 4.60E-134 | PL1_4(112-295) | PL1 | PL1_4 |
| cluster43 | 3 | FOMED\|g12671.t1  ;FOP\|EXA33957.1;  FOC\|g9658.t1; | Q96UT0 | Cutinase 2 | GO:0005576; C:extracellular region; IEA:UniProtKB-KW; GO:0050525; F:cutinase activity; IEA:UniProtKB-EC | 1.80E-96 | CE5(47-223) | CE5 | CE5 |
| cluster70 | 3 | FOMED\|g13776.t1;  FOP\|EXA43993.1;  FOC\|g8742.t1; | Q99174 | Cutinase | GO:0005576; C:extracellular region; IEA:UniProtKB-KW; GO:0050525; F:cutinase activity; IEA:UniProtKB-EC | 1.00E-94 | CE5(52-228) | CE5 | CE5 |
| cluster58 | 3 | FOMED\|g14776.t1;  FOP\|EXA30199.1  ;FOC\|g8371.t1; | O74641 | Extracellular endo-inulinase inuA | GO:0005576; C:extracellular region; IEA:UniProtKB-KW; GO:0051670; F:inulinase activity; IEA:UniProtKB-EC; GO:0000272; P:polysaccharide catabolic process; IEA:UniProtKB-KW | 8.50E-203 | GH32(41-364) | GH32 | GH32 |
| cluster87 | 3 | FOMED\|g1362.t1;  FOP\|EXA41616.1;  FOC\|g10025.t1; | Q99172 | Alpha-galactosidase | GO:0005576; C:extracellular region; IEA:UniProtKB-KW; GO:0052692; F:raffinose alpha-galactosidase activity; IEA:UniProtKB-EC; GO:0005975; P:carbohydrate metabolic process; IEA:InterPro | 5.80E-82 | GH27(114-338) | GH27 | GH27 |
| cluster4 | 5 | FOP\|EXA43881.1;  FOP\|EXA43880.1;F  OP\|EXA43882.1;  FOMED\|g6468.t1;  FOC\|g6255.t1; | O94221 | Probable alpha-galactosidase B | GO:0005576; C:extracellular region; IEA:UniProtKB-KW; GO:0052692; F:raffinose alpha-galactosidase activity; IEA:UniProtKB-EC; GO:0005975; P:carbohydrate metabolic process; IEA:InterPro | 4.10E-83 | N | GH27 | GH27 |
| cluster127 | 3 | FOMED\|g9424.t1;  FOP\|EXA35717.1;  FOC\|g11374.t1; | A1D145 | Probable endopolygalacturonase NFIA_008150 | GO:0005576; C:extracellular region; ISS:UniProtKB; GO:0004650; F:polygalacturonase activity; ISS:UniProtKB; GO:0071555; P:cell wall organization; IEA:UniProtKB-KW; GO:0045490; P:pectin catabolic process; ISS:UniProtKB | 7.90E-142 | GH28(54-376) | GH28 | GH28 |
| cluster24 | 3 | FOMED\|g12468.t1  ;FOP\|EXA34830.1;  FOC\|g8257.t1; | Q4WL66 | Probable alpha-L-arabinofuranosidase B | GO:0005576; C:extracellular region; ISS:UniProtKB; GO:0046556; F:alpha-L-arabinofuranosidase activity; ISS:UniProtKB; GO:0031222; P:arabinan catabolic process; IEA:UniProtKB-UniPathway; GO:0019566; P:arabinose metabolic process; ISS:UniProtKB; GO:0046373; P:L-arabinose metabolic process; IEA:InterPro; GO:0045493; P:xylan catabolic process; IEA:UniProtKB-KW | 2.20E-232 | GH54(22-336)+CBM42(354-494) | GH54+CBM42 | CBM42 |
| cluster122 | 3 | FOMED\|g6083.t1  ;FOP\|EXA43241.1;  FOC\|g9844.t1; | Q5AVN4 | Pectate lyase A | GO:0005576; C:extracellular region; ISS:UniProtKB; GO:0046872; F:metal ion binding; IEA:UniProtKB-KW; GO:0030570; F:pectate lyase activity; IDA:UniProtKB; GO:0071555; P:cell wall organization; IEA:UniProtKB-KW; GO:0045490; P:pectin catabolic process; IDA:UniProtKB | 1.20E-53 | PL1_10(80-253) | PL1 | PL1_10 |
| cluster113 | 3 | FOMED\|g10130.t1;  FOP\|EXA36849.1;  FOC\|g9100.t1; | B0YAA4 | Probable exopolygalacturonase B | GO:0005576; C:extracellular region; ISS:UniProtKB; GO:0047911; F:galacturan 1,4-alpha-galacturonidase activity; IEA:UniProtKB-EC; GO:0004650; F:polygalacturonase activity; ISS:UniProtKB; GO:0071555; P:cell wall organization; IEA:UniProtKB-KW; GO:0045490; P:pectin catabolic process; ISS:UniProtKB | 5.20E-100 | GH28(125-398) | GH28 | GH28 |
| cluster40 | 3 | FOMED\|g10592.t1;  FOP\|EXA35360.1;  FOC\|g7660.t1; | Q12535 | Pectinesterase | GO:0005618; C:cell wall; IEA:InterPro; GO:0005576; C:extracellular region; IEA:UniProtKB-KW; GO:0045330; F:aspartyl esterase activity; IEA:UniProtKB-KW; GO:0030599; F:pectinesterase activity; IEA:UniProtKB-EC; GO:0042545; P:cell wall modification; IEA:InterPro; GO:0045490; P:pectin catabolic process; IEA:UniProtKB-UniPathway | 2.50E-91 | CE8(27-306) | CE8 | CE8 |
| cluster106 | 3 | FOMED\|g13169.t1;  FOP\|EXA51027.1;  FOC\|g7881.t1; | P17872 | Pectinesterase | GO:0005618; C:cell wall; IEA:InterPro; GO:0005576; C:extracellular region; IEA:UniProtKB-KW; GO:0045330; F:aspartyl esterase activity; IEA:UniProtKB-KW; GO:0030599; F:pectinesterase activity; IEA:UniProtKB-EC; GO:0042545; P:cell wall modification; IEA:InterPro; GO:0045490; P:pectin catabolic process; IEA:UniProtKB-UniPathway | 5.20E-14 | CE8(51-381) | CE8 | CE8 |
| cluster10 | 4 | FOP\|EXA54007.1;  FOP\|EXA54008.1;  FOMED\|g1285.t1  ;FOC\|g1523.t1; | Q4WGL5 | Secreted beta-glucosidase sun1 | GO:0005618; C:cell wall; IEA:UniProtKB-KW; GO:0005576; C:extracellular region; IEA:UniProtKB-KW; GO:0030246; F:carbohydrate binding; IDA:ASPGD; GO:0016798; F:hydrolase activity, acting on glycosyl bonds; IEA:UniProtKB-KW; GO:0006076; P:(1->3)-beta-D-glucan catabolic process; IDA:ASPGD; GO:0071555; P:cell wall organization; IEA:UniProtKB-KW; GO:0030448; P:hyphal growth; IMP:ASPGD | 4.80E-90 | GH132(137-438) | GH132 | GH132 |
| cluster11 | 4 | FOMED\|g11788.t1  ;FOP\|EXA35249.1;  FOC\|g13623.t1;  FOP\|EXA34284.1; | O13716 | Glucan endo-1,3-alpha-glucosidase agn1 | GO:0005618; C:cell wall; IEA:UniProtKB-KW; GO:0005576; C:extracellular region; IEA:UniProtKB-KW; GO:0051118; F:glucan endo-1,3-alpha-glucosidase activity; IEA:UniProtKB-EC; GO:0007049; P:cell cycle; IEA:UniProtKB-KW; GO:0051301; P:cell division; IEA:UniProtKB-KW; GO:0071555; P:cell wall organization; IEA:UniProtKB-KW | 8.40E-46 | GH71(21-411)+CBM24(456-530)+CBM24(555-630) | GH71 | CBM24 |
| cluster35 | 3 | FOMED\|g7561.t1;  FOP\|EXA38492.1;  FOC\|g7366.t1; | Q06702 | Chitin deacetylase 1 | GO:0005631; C:chitosan layer of spore wall; ISS:SGD; GO:0004099; F:chitin deacetylase activity; IDA:SGD; GO:0030476; P:ascospore wall assembly; IDA:SGD; GO:0006032; P:chitin catabolic process; IEA:UniProtKB-KW; GO:0000272; P:polysaccharide catabolic process; IEA:UniProtKB-KW | 4.00E-18 | CE4(32-155) | CE4 | CE4 |
| cluster53 | 3 | FOMED\|g11221.t1;  FOP\|EXA39543.1;  FOC\|g5146.t1; | O42970 | Uncharacterized serine-rich protein C1E8.05 | GO:0005739; C:mitochondrion; IDA:PomBase | 0.00065 | CBM63(232-302) | CBM63 | CBM63 |
| cluster45 | 3 | FOMED\|g12369.t1;  FOP\|EXA34914.1;  FOC\|g10904.t1; | A6REI4 | Putative lipase ATG15 | GO:0005783; C:endoplasmic reticulum; IEA:UniProtKB-KW; GO:0005768; C:endosome; IEA:UniProtKB-KW; GO:0005794; C:Golgi apparatus; IEA:UniProtKB-KW; GO:0016021; C:integral component of membrane; IEA:UniProtKB-KW; GO:0004806; F:triglyceride lipase activity; IEA:UniProtKB-EC; GO:0006914; P:autophagy; IEA:UniProtKB-KW; GO:0016042; P:lipid catabolic process; IEA:UniProtKB-KW | 0.0027 | AA11(20-211) | AA11 | AA11 |
| cluster65 | 3 | FOMED\|g10240.t1;  FOP\|EXA36714.1;  FOC\|g7164.t1; | Q12062 | Versicolorin B synthase | GO:0005829; C:cytosol; TAS:UniProtKB; GO:0008812; F:choline dehydrogenase activity; IEA:InterPro; GO:0050660; F:flavin adenine dinucleotide binding; IEA:InterPro; GO:0016836; F:hydro-lyase activity; IDA:UniProtKB; GO:0042803; F:protein homodimerization activity; IDA:UniProtKB; GO:0045122; P:aflatoxin biosynthetic process; IDA:UniProtKB; GO:0006066; P:alcohol metabolic process; IEA:InterPro | 2.10E-126 | AA3_2(64-639) | AA3+AA8 | AA3_2 |
| cluster102 | 3 | FOMED\|g9408.t1;  FOP\|EXA35739.1;  FOC\|g8124.t1; | Q1E3R8 | Endochitinase 1 | GO:0008061; F:chitin binding; IEA:UniProtKB-KW; GO:0004568; F:chitinase activity; IEA:UniProtKB-EC; GO:0006032; P:chitin catabolic process; IEA:UniProtKB-KW; GO:0000272; P:polysaccharide catabolic process; IEA:UniProtKB-KW | 1.30E-28 | GH18(49-404) | N | GH18 |
| cluster72 | 3 | FOMED\|g12144.t1;  FOP\|EXA51173.1;  FOC\|g8935.t1; | P09805 | Killer toxin subunits alpha/beta | GO:0008061; F:chitin binding; IEA:UniProtKB-KW; GO:0004568; F:chitinase activity; IEA:UniProtKB-EC; GO:0006032; P:chitin catabolic process; IEA:UniProtKB-KW; GO:0000272; P:polysaccharide catabolic process; IEA:UniProtKB-KW | 9.60E-27 | GH18(85-412) | N | CBM18 |
| cluster77 | 3 | FOMED\|g1584.t1;  FOP\|EXA41331.1;  FOC\|g14562.t1; | P46237 | Endoglucanase type C | GO:0008810; F:cellulase activity; IEA:UniProtKB-EC; GO:0030245; P:cellulose catabolic process; IEA:UniProtKB-KW | 2.10E-260 | GH7(22-416) | GH7 | GH7 |
| cluster28 | 3 | FOMED\|g12488.t1;  FOP\|EXA34806.1;  FOC\|g8279.t1; | P0C1B3 | Alpha-amylase A type-1/2 | GO:0030428; C:cell septum; IDA:ASPGD; GO:0030287; C:cell wall-bounded periplasmic space; IDA:ASPGD; GO:0005576; C:extracellular region; IDA:ASPGD; GO:0009277; C:fungal-type cell wall; IDA:ASPGD; GO:0032163; C:hyphal septin band; IDA:ASPGD; GO:0031521; C:spitzenkorper; IDA:ASPGD; GO:0004556; F:alpha-amylase activity; IGI:ASPGD; GO:0005509; F:calcium ion binding; IEA:InterPro; GO:0016052; P:carbohydrate catabolic process; IGI:ASPGD | 7.20E-105 | GH13_1(58-345) | N | GH13_1 |
| cluster32 | 3 | FOMED\|g6680.t1;  FOP\|EXA48756.1;  FOC\|g10590.t1; | Q8J0P4 | Probable glycosidase crf1 | GO:0031225; C:anchored component of membrane; IEA:UniProtKB-KW; GO:0005576; C:extracellular region; IEA:UniProtKB-KW; GO:0009277; C:fungal-type cell wall; IDA:ASPGD; GO:0005886; C:plasma membrane; IEA:UniProtKB-KW; GO:0004553; F:hydrolase activity, hydrolyzing O-glycosyl compounds; IEA:InterPro; GO:0019863; F:IgE binding; IDA:ASPGD; GO:0005975; P:carbohydrate metabolic process; IEA:InterPro; GO:0071555; P:cell wall organization; IEA:UniProtKB-KW | 2.00E-70 | GH16(78-219) | GH16 | GH16 |
| cluster83 | 3 | FOMED\|g6701.t1;  FOP\|EXA43123.1;  FOC\|g8982.t1; | Q92223 | Endochitinase A | GO:0031225; C:anchored component of membrane; IEA:UniProtKB-KW; GO:0005618; C:cell wall; IEA:UniProtKB-KW; GO:0005576; C:extracellular region; IEA:UniProtKB-KW; GO:0005886; C:plasma membrane; IEA:UniProtKB-KW; GO:0008061; F:chitin binding; IEA:UniProtKB-KW; GO:0004568; F:chitinase activity; IEA:UniProtKB-EC; GO:0006032; P:chitin catabolic process; IEA:UniProtKB-KW; GO:0000272; P:polysaccharide catabolic process; IEA:UniProtKB-KW | 0.00035 | CE3(30-220) | CE3 | CE3 |
| cluster82 | 3 | FOMED\|g2640.t1;  FOP\|EXA49904.1;  FOC\|g4337.t1; | Q4X084 | Probable endo-1,3(4)-beta-glucanase AFUA_2G14360 | GO:0031225; C:anchored component of membrane; IEA:UniProtKB-KW; GO:0005886; C:plasma membrane; IEA:UniProtKB-KW; GO:0052861; F:glucan endo-1,3-beta-glucanase activity, C-3 substituted reducing group; IDA:ASPGD; GO:0052862; F:glucan endo-1,4-beta-glucanase activity, C-3 substituted reducing group; IEA:UniProtKB-EC; GO:0030245; P:cellulose catabolic process; IEA:UniProtKB-KW | 2.10E-76 | GH16(83-319) | GH16 | GH16 |
| cluster69 | 3 | FOMED\|g8243.t1;  FOP\|EXA46209.1;  FOC\|g10511.t1; | Q2GVT8 | Protein transport protein SEC31 | GO:0031410; C:cytoplasmic vesicle; IEA:UniProtKB-KW; GO:0005783; C:endoplasmic reticulum; IEA:UniProtKB-KW; GO:0016020; C:membrane; IEA:UniProtKB-KW; GO:0015031; P:protein transport; IEA:UniProtKB-KW; GO:0016192; P:vesicle-mediated transport; IEA:UniProtKB-KW | 3.90E-05 | AA11(21-227) | AA11 | AA11 |

**Supplementary table S5:** Orthologue clusters of CAZymes with GO annotation**.**(FOMED: f. sp. *medicaginis*, FOP: f. sp. *pisi*, FOC: f. sp. *ciceris*) **FOMED:** *F.oxysporum* f. sp. *medicaginis***, FOC:** *F. oxysporum* f. sp. *ciceris***, FOP:** *F. oxysporum* f. sp. *pisi* **;
AA:** Auxiliary Activities, **CE**: Carbohydrate Esterase, **CBM**: Carbohydrate Binding Module, **GH**: Glycosyl hydrolase, **PL**: Polysaccharide lyase

| **Accessions** | **Protein accessions** | **LogFC** | | **HMMER** | **DIAMOND** | **HotPep** | **SignalP** |
| --- | --- | --- | --- | --- | --- | --- | --- |
|  |  | **Fomed vs. Fol** | **Fop vs. Fol** |  |  |  |  |
| FOXG_00135 | XP_018231794.1 | -2.43290908 | -2.65255005 | N | GH17 | GH17 | SP(Sec/SPI) |
| FOXG_06344 | XP_018242147.1 | 2.646513249 | 2.901413256 | AA1_3(51-365) | AA1 | AA1_3 | SP(Sec/SPI) |
| FOXG_13227 | XP_018252396.1 | 8.09872351 | 7.012390122 | AA1_3(90-321) | AA1 | AA1 | OTHER |
| FOXG_14565 | XP_018254143.1 | 2.586016691 | 5.502733486 | AA1_3(96-418) | AA1 | AA1_3 | SP(Sec/SPI) |
| FOXG_13185 | XP_018252346.1 | -3.892114923 | 7.635479262 | AA1_3(96-426) | AA1 | AA1_3 | SP(Sec/SPI) |
| FOXG_07797 | XP_018243596.1 | -4.570098596 | -3.81735737 | AA11(18-208) | AA11 | AA11 | SP(Sec/SPI) |
| FOXG_13164 | XP_018252324.1 | -4.330754874 | -2.07258368 | AA3_2(23-596) | AA3+AA8 | AA3_2 | SP(Sec/SPI) |
| FOXG_14236 | XP_018253950.1 | -4.84604374 | -3.33793958 | AA3_2(33-607) | AA3 | AA3_2 | SP(Sec/SPI) |
| FOXG_12395 | XP_018250966.1 | 5.73916094 | 5.721168125 | AA3_2(5-546) | AA3 | AA3_2 | OTHER |
| FOXG_13301 | XP_018252486.1 | -9.181644633 | -4.00894045 | AA3_2(64-639) | AA3+AA8 | AA3_2 | SP(Sec/SPI) |
| FOXG_03204 | XP_018237170.1 | 3.87192596 | 6.534118114 | AA3_2(7-575) | AA3 | AA3_2 | OTHER |
| FOXG_09652 | XP_018247017.1 | 6.394052568 | 7.549749749 | AA3_3(12-603) | AA3 | AA3_3 | OTHER |
| FOXG_01379 | XP_018234068.1 | 5.660557601 | 4.960205203 | AA4(25-546) | N | AA4 | OTHER |
| FOXG_13095 | XP_018252222.1 | -7.938680015 | -4.38450739 | AA5(390-895) | AA5 | AA5_1 | SP(Sec/SPI) |
| FOXG_08903 | XP_018245899.1 | -6.928852788 | -6.1962148 | AA5_2(96-708) | AA5 | AA5_2 | SP(Sec/SPI) |
| FOXG_03535 | XP_018237763.1 | -3.171933421 | -3.85276471 | AA7(52-263) | N | AA7 | SP(Sec/SPI) |
| FOXG_07821 | XP_018243640.1 | -2.084621094 | -2.50272716 | AA9(112-224) | AA9 | AA9 | SP(Sec/SPI) |
| FOXG_04019 | XP_018238497.1 | -9.425589689 | -5.43947116 | AA9(5-135) | AA9 | N | SP(Sec/SPI) |
| FOXG_08211 | XP_018244804.1 | -10.30713334 | -7.63951723 | AA9(7-222) | AA9 | AA9 | SP(Sec/SPI) |
| FOXG_14505 | XP_018254072.1 | -7.808104861 | -6.17702355 | AA9(8-228) | AA9 | AA9 | SP(Sec/SPI) |
| FOXG_10845 | XP_018248753.1 | -4.131272287 | -2.42652464 | CBM67(128-293)+GH78(324-843) | GH78 | GH78 | OTHER |
| FOXG_04665 | XP_018239456.1 | 2.832572754 | 8.861776701 | CBM67(128-302)+GH78(332-862) | GH78+CBM67 | GH78 | OTHER |
| FOXG_11916 | XP_018250337.1 | 2.676423644 | 2.958315539 | CBM67(136-305)+GH78(332-857) | GH78+CBM67 | GH78 | OTHER |
| FOXG_17228 | XP_018258161.1 | -5.618913399 | -5.42503345 | CBM67(147-331)+GH78(357-881) | GH78+CBM67 | GH78 | OTHER |
| FOXG_09633 | XP_018246993.1 | 3.596779947 | 4.689533584 | CE16(33-282) | N | CE16 | SP(Sec/SPI) |
| FOXG_04056 | XP_018238541.1 | -3.106113606 | 4.447616241 | CE2(128-339) | CE2 | CE2 | SP(Sec/SPI) |
| FOXG_21586 | XP_018254196.1 | -5.165353311 | 4.586759043 | CE3(90-280) | CE3 | CE3 | SP(Sec/SPI) |
| FOXG_09221 | XP_018246303.1 | -13.72593483 | -10.1846896 | CE4(32-155) | CE4 | CE4 | SP(Sec/SPI) |
| FOXG_14254 | XP_018253977.1 | -6.222021786 | -5.93353225 | GH125(86-496) | GH125 | GH125 | SP(Sec/SPI) |
| FOXG_04533 | XP_018239301.1 | -3.722039696 | -4.19209778 | GH128(43-275) | N | GH128 | SP(Sec/SPI) |
| FOXG_04689 | XP_018239494.1 | -11.01843001 | -8.29444623 | GH13_15(353-578) | N | CBM21 | SP(Sec/SPI) |
| FOXG_14959 | XP_018255008.1 | -8.886508769 | -7.195411 | GH13_40(36-409) | GH13 | GH13_40 | OTHER |
| FOXG_17416 | XP_018258404.1 | 3.817237856 | 3.806294449 | GH142(20-492) | N | CBM13 | OTHER |
| FOXG_14735 | XP_018254357.1 | -5.000206928 | -2.64871097 | GH16(115-442) | N | GH16 | OTHER |
| FOXG_13096 | XP_018252223.1 | -8.32743503 | -6.43535775 | GH16(129-468) | GH16 | GH16 | OTHER |
| FOXG_16869 | XP_018257676.1 | -3.850567019 | -2.70650839 | GH16(49-259) | N | GH16 | SP(Sec/SPI) |
| FOXG_09759 | XP_018247162.1 | 2.307710117 | 5.516693694 | GH16(82-312) | N | GH16 | SP(Sec/SPI) |
| FOXG_22639 | XP_018257788.1 | -11.9735688 | -12.0999312 | GH18(115-462) | GH18 | CBM18 | SP(Sec/SPI) |
| FOXG_14329 | XP_018254522.1 | -9.920658152 | -8.16839139 | GH18(117-464) | GH18+CBM18 | CBM18 | SP(Sec/SPI) |
| FOXG_15151 | XP_018255725.1 | -6.619910898 | -4.81967675 | GH18(117-464) | GH18+CBM18 | CBM18 | SP(Sec/SPI) |
| FOXG_22648 | XP_018257851.1 | -4.921540108 | -3.9334275 | GH18(228-553) | CBM18 | CBM18 | SP(Sec/SPI) |
| FOXG_14840 | XP_018254813.1 | -12.23689862 | -10.3174333 | GH18(34-391) | GH18 | GH18 | OTHER |
| FOXG_16251 | XP_018256878.1 | -7.481910753 | -6.61906145 | GH18(34-391) | GH18 | GH18 | OTHER |
| FOXG_15373 | XP_018255330.1 | -3.693627364 | -8.73634306 | GH18(520-874) | GH18+CBM18+CBM50 | CBM18 | SP(Sec/SPI) |
| FOXG_11492 | XP_018249715.1 | -3.864896676 | 4.142568932 | GH18(5-336) | N | GH18 | OTHER |
| FOXG_17685 | XP_018258804.1 | -10.12357894 | -8.090211 | GH18(562-908)+CBM24(927-1004) | GH18+CBM18+CBM50 | GH18 | SP(Sec/SPI) |
| FOXG_02594 | XP_018236228.1 | 2.400018593 | 3.857725655 | GH2(4-741) | GH2 | GH2 | OTHER |
| FOXG_12895 | XP_018251424.1 | 2.84185557 | 6.937365829 | GH20(169-509) | GH20 | GH20 | SP(Sec/SPI) |
| FOXG_17226 | XP_018258159.1 | -3.047312795 | -4.5668306 | GH20(172-496) | GH20 | GH20 | SP(Sec/SPI) |
| FOXG_08862 | XP_018245843.1 | 3.310468839 | 4.506068596 | GH28(83-437) | GH28 | GH28 | SP(Sec/SPI) |
| FOXG_12336 | XP_018250892.1 | 4.413215815 | 3.1780714 | GH3(37-248) | GH3 | GH3 | OTHER |
| FOXG_02734 | XP_018236411.1 | -3.189441073 | -5.56979516 | GH3(68-284) | GH3 | GH3 | SP(Sec/SPI) |
| FOXG_15250 | XP_018255170.1 | -5.550987991 | -5.81966214 | GH3(69-273) | GH3 | GH3 | OTHER |
| FOXG_02663 | XP_018236326.1 | 2.456515278 | 7.682447969 | GH31(292-533) | N | GH31 | SP(Sec/SPI) |
| FOXG_18378 | XP_018236327.1 | 3.052567679 | 5.538594255 | GH31(6-260) | N | GH31 | OTHER |
| FOXG_17514 | XP_018258527.1 | -5.716050839 | -7.85431145 | GH32(1-260) | N | CBM38 | OTHER |
| FOXG_17516 | XP_018258530.1 | 3.674176516 | 5.277712955 | GH32(19-364) | GH32 | GH32 | OTHER |
| FOXG_11757 | XP_018250131.1 | 5.174763519 | 4.361854605 | GH32(30-323)+CBM38(333-432) | GH32 | CBM38 | SP(Sec/SPI) |
| FOXG_04590 | XP_018239367.1 | 4.841356958 | 5.830686174 | GH33(21-321) | N | GH33 | OTHER |
| FOXG_04098 | XP_018238607.1 | 4.024090751 | 3.18598083 | GH43_13(9-315) | GH43 | GH43_13 | OTHER |
| FOXG_12990 | XP_018251546.1 | -2.737621305 | -5.82243811 | GH43_14(7-305) | N | GH43_14 | OTHER |
| FOXG_08235 | XP_018244847.1 | -2.256660719 | -3.62898284 | GH43_21(214-442) | N | GH43_21 | OTHER |
| FOXG_04368 | XP_018239065.1 | 4.347900946 | 3.122799621 | GH43_26(21-316) | N | GH43_26 | SP(Sec/SPI) |
| FOXG_09629 | XP_018246989.1 | -2.521270734 | -2.70134135 | GH43_36(30-300) | GH43 | GH43_36 | SP(Sec/SPI) |
| FOXG_15355 | XP_018255303.1 | -3.298281224 | -2.75401286 | GH49(25-598) | GH49 | GH49 | OTHER |
| FOXG_10041 | XP_018247517.1 | -4.439848681 | -2.30644701 | GH5_15(82-384) | GH5 | GH5_15 | SP(Sec/SPI) |
| FOXG_13229 | XP_018252399.1 | 3.622302324 | 5.246606956 | GH5_16(34-373) | GH5 | GH5_16 | SP(Sec/SPI) |
| FOXG_14629 | XP_018254219.1 | 7.896808899 | 4.267728893 | GH5_31(47-313) | N | GH5 | SP(Sec/SPI) |
| FOXG_19189 | XP_018241799.1 | -2.011979909 | -3.5366135 | GH55(17-754) | GH55 | GH55 | SP(Sec/SPI) |
| FOXG_16409 | XP_018257304.1 | -5.86145446 | -5.67682575 | GH55(2-665) | GH55 | GH55 | OTHER |
| FOXG_10710 | XP_018248584.1 | 3.283141441 | 4.229375966 | GH64(69-441) | GH64 | GH64 | SP(Sec/SPI) |
| FOXG_21794 | XP_018254819.1 | -10.28683324 | -8.49646152 | GH64(75-445) | N | GH64 | SP(Sec/SPI) |
| FOXG_12407 | XP_018251649.1 | -11.50554936 | -9.67406223 | GH64(76-449) | GH64 | GH64 | SP(Sec/SPI) |
| FOXG_14130 | XP_018253745.1 | -9.260048339 | -9.16088532 | GH64(76-449) | GH64 | GH64 | SP(Sec/SPI) |
| FOXG_09745 | XP_018247147.1 | -3.388811803 | -4.75484872 | GH76(32-325) | N | GH76 | OTHER |
| FOXG_13640 | XP_018252931.1 | -5.126258882 | -3.71984149 | GH76(34-415) | GH76 | GH76 | SP(Sec/SPI) |
| FOXG_00357 | XP_018232260.1 | 3.144945137 | 3.395589124 | GH78(404-810) | GH78 | GH78 | SP(Sec/SPI) |
| FOXG_11759 | XP_018250133.1 | 4.246457299 | 6.158526222 | GH79(97-453) | GH79 | N | SP(Sec/SPI) |
| FOXG_03402 | XP_018237519.1 | -7.978187831 | -9.54580193 | N | GH18 | GH18 | OTHER |
| FOXG_10087 | XP_018247567.1 | -4.967591455 | 2.366495625 | N | GH39 | GH39 | SP(Sec/SPI) |
| FOXG_16879 | XP_018257690.1 | 7.769539178 | 5.504589503 | N | AA1 | AA1 | OTHER |
| FOXG_13331 | XP_018252524.1 | -6.356431176 | -5.44182293 | PL1_4(108-294) | N | CBM1 | SP(Sec/SPI) |
| FOXG_16516 | XP_018257112.1 | 2.840385858 | 5.402794889 | PL1_4(113-297) | PL1 | PL1_4 | SP(Sec/SPI) |
| FOXG_05948 | XP_018241515.1 | -2.970774165 | -4.77238656 | PL1_7(79-260) | PL1 | PL1_7 | SP(Sec/SPI) |
| FOXG_13801 | XP_018253145.1 | -5.903513056 | -4.12505349 | PL1_9(83-263) | PL1 | PL1_9 | SP(Sec/SPI) |
| FOXG_02656 | XP_018236318.1 | -2.91526667 | -4.75788297 | PL11_2(22-594) | PL11 | CBM13 | SP(Sec/SPI) |
| FOXG_12312 | XP_018250863.1 | -5.318429376 | -5.70726266 | PL9_3(18-377) | PL9 | PL9_3 | SP(Sec/SPI) |

**Supplementary table S6:** Differentially expressed CAZymes with presence and absence of signal peptides (Accessions in green are up-regulated, accessions in red are down-regulated and accessions in black showing difference in expression pattern in two comparisons**). FOMED:** *F.oxysporum* f. sp. *medicaginis***, FOL:** *F. oxysporum* f. sp. *lycopersici***, FOP:** *F. oxysporum* f. sp. *pisi* **; AA:** Auxiliary Activities, **CE**: Carbohydrate Esterase, **CBM**: Carbohydrate Binding Module, **GH**: Glycosyl hydrolase, **PL**: Polysaccharide lyase

| Entry ID | Localization | Type | Nucleus | Cytoplasm | Extra-cellular | Mitochondrion | Cell membrane | Endoplasmic reticulum | Plastid | Golgi apparatus | Lysosome/  Vacuole | Peroxisome |
| --- | --- | --- | --- | --- | --- | --- | --- | --- | --- | --- | --- | --- |
| XP_018239301.1 | Cell membrane | Membrane | 0.0006 | 0.0002 | 0.0049 | 0.0005 | 0.8515 | 0.066 | 0.0005 | 0.0095 | 0.0663 | 0.0002 |
| XP_018252931.1 | Cell membrane | Membrane | 0 | 0.0003 | 0.2212 | 0.005 | 0.5553 | 0.0337 | 0.0006 | 0.002 | 0.1815 | 0.0003 |
| XP_018238497.1 | Extracellular | Soluble | 0 | 0.0006 | 0.9941 | 0.0001 | 0.0001 | 0.0007 | 0 | 0 | 0.0045 | 0 |
| XP_018243640.1 | Extracellular | Soluble | 0.0001 | 0.0031 | 0.705 | 0.0007 | 0.0041 | 0.0333 | 0.0001 | 0.0032 | 0.2502 | 0 |
| XP_018257676.1 | Extracellular | Soluble | 0.0005 | 0.0089 | 0.8808 | 0.0219 | 0.0014 | 0.0314 | 0.007 | 0.0002 | 0.0477 | 0.0002 |
| XP_018246303.1 | Extracellular | Soluble | 0.0011 | 0.0035 | 0.8252 | 0.0141 | 0.0008 | 0.1246 | 0.0008 | 0.0001 | 0.0295 | 0.0002 |
| XP_018246993.1 | Extracellular | Soluble | 0.0003 | 0.0169 | 0.762 | 0.0024 | 0.0006 | 0.0271 | 0.0002 | 0.0002 | 0.1893 | 0.0011 |
| XP_018253145.1 | Extracellular | Soluble | 0 | 0.001 | 0.9571 | 0.0005 | 0.0002 | 0.0217 | 0.0003 | 0 | 0.0191 | 0 |
| XP_018241515.1 | Extracellular | Soluble | 0 | 0.0004 | 0.9927 | 0.0015 | 0.0001 | 0.0007 | 0 | 0 | 0.0047 | 0 |
| XP_018244804.1 | Extracellular | Soluble | 0 | 0.0002 | 0.9989 | 0.0001 | 0.0002 | 0.0003 | 0 | 0 | 0.0003 | 0 |
| XP_018239065.1 | Extracellular | Soluble | 0.0001 | 0.004 | 0.8712 | 0.001 | 0.0012 | 0.0223 | 0.0003 | 0.0001 | 0.0999 | 0 |
| XP_018247162.1 | Extracellular | Soluble | 0 | 0.0001 | 0.9891 | 0 | 0 | 0.0002 | 0 | 0 | 0.0106 | 0 |
| XP_018238541.1 | Extracellular | Soluble | 0.0002 | 0.0268 | 0.782 | 0.0735 | 0.0009 | 0.0277 | 0.0333 | 0.0001 | 0.0461 | 0.0094 |
| XP_018254072.1 | Extracellular | Soluble | 0.0014 | 0.0002 | 0.9977 | 0 | 0.0002 | 0.0003 | 0 | 0 | 0.0002 | 0 |
| XP_018254219.1 | Extracellular | Soluble | 0 | 0 | 0.9875 | 0 | 0 | 0.0008 | 0 | 0 | 0.0117 | 0 |
| XP_018257112.1 | Extracellular | Soluble | 0 | 0 | 0.9971 | 0 | 0 | 0.0007 | 0 | 0 | 0.0022 | 0 |
| XP_018250863.1 | Extracellular | Soluble | 0 | 0.0003 | 0.9937 | 0.0003 | 0.0007 | 0.002 | 0 | 0 | 0.0031 | 0 |
| XP_018243596.1 | Extracellular | Soluble | 0.0002 | 0 | 0.9993 | 0.0001 | 0.0001 | 0.0002 | 0 | 0 | 0.0001 | 0 |
| XP_018247517.1 | Extracellular | Soluble | 0 | 0.0006 | 0.8943 | 0.0001 | 0 | 0.0083 | 0 | 0 | 0.0967 | 0 |
| XP_018247567.1 | Extracellular | Soluble | 0 | 0 | 0.9963 | 0 | 0 | 0.0005 | 0 | 0 | 0.0032 | 0 |
| XP_018248584.1 | Extracellular | Soluble | 0 | 0.0001 | 0.7729 | 0.0001 | 0 | 0.0072 | 0 | 0 | 0.2197 | 0 |
| XP_018245843.1 | Extracellular | Soluble | 0.0001 | 0.0024 | 0.8739 | 0.0047 | 0.0035 | 0.0235 | 0.002 | 0.0021 | 0.0879 | 0 |
| XP_018254819.1 | Extracellular | Soluble | 0.0001 | 0.0011 | 0.7311 | 0.0008 | 0.0009 | 0.0742 | 0.0003 | 0.0001 | 0.1913 | 0.0001 |
| XP_018253745.1 | Extracellular | Soluble | 0 | 0.0009 | 0.9045 | 0.0011 | 0.0006 | 0.0107 | 0.0002 | 0.0002 | 0.0817 | 0 |
| XP_018251649.1 | Extracellular | Soluble | 0 | 0.0009 | 0.906 | 0.0011 | 0.0006 | 0.0105 | 0.0002 | 0.0002 | 0.0804 | 0.0001 |
| XP_018237763.1 | Extracellular | Soluble | 0.002 | 0.031 | 0.5692 | 0.2302 | 0.002 | 0.047 | 0.0069 | 0.005 | 0.0764 | 0.0304 |
| XP_018250133.1 | Extracellular | Soluble | 0.0001 | 0.0124 | 0.9351 | 0.0254 | 0.001 | 0.0066 | 0.0016 | 0.0001 | 0.0134 | 0.0044 |
| XP_018253977.1 | Extracellular | Soluble | 0.0001 | 0.0014 | 0.9261 | 0.0024 | 0.0003 | 0.0173 | 0.0001 | 0.0001 | 0.0521 | 0.0001 |
| XP_018246989.1 | Extracellular | Soluble | 0 | 0.0002 | 0.9956 | 0.0001 | 0 | 0.0003 | 0 | 0 | 0.0038 | 0 |
| XP_018252524.1 | Extracellular | Soluble | 0.0002 | 0.0007 | 0.9938 | 0.0009 | 0.0004 | 0.0022 | 0 | 0.0001 | 0.0018 | 0 |
| XP_018242147.1 | Extracellular | Soluble | 0.0001 | 0.0013 | 0.8766 | 0.0002 | 0.0003 | 0.0479 | 0 | 0 | 0.0736 | 0 |
| XP_018236326.1 | Extracellular | Soluble | 0 | 0.0003 | 0.7403 | 0.0002 | 0.0012 | 0.1531 | 0 | 0 | 0.1048 | 0 |
| XP_018252324.1 | Extracellular | Soluble | 0 | 0.0063 | 0.9534 | 0.0059 | 0.0003 | 0.0182 | 0.0001 | 0 | 0.015 | 0.0007 |
| XP_018236318.1 | Extracellular | Soluble | 0.0002 | 0.0043 | 0.7598 | 0.0015 | 0.0002 | 0.0448 | 0.0005 | 0 | 0.1887 | 0.0001 |
| XP_018253950.1 | Extracellular | Soluble | 0 | 0.0005 | 0.9552 | 0.0003 | 0.0003 | 0.0288 | 0 | 0 | 0.0149 | 0 |
| XP_018231794.1 | Extracellular | Soluble | 0.005 | 0.0112 | 0.9677 | 0.0093 | 0.0038 | 0.0009 | 0.0007 | 0 | 0.0015 | 0 |
| XP_018252346.1 | Extracellular | Soluble | 0.0002 | 0.0004 | 0.6754 | 0.0015 | 0.0086 | 0.103 | 0.0001 | 0.0006 | 0.2102 | 0 |
| XP_018254143.1 | Extracellular | Soluble | 0 | 0.0004 | 0.966 | 0.0001 | 0.0002 | 0.0031 | 0 | 0 | 0.0301 | 0 |
| XP_018252486.1 | Extracellular | Soluble | 0.0011 | 0.0274 | 0.7749 | 0.0519 | 0.0006 | 0.0413 | 0.0186 | 0 | 0.0815 | 0.0026 |
| XP_018250131.1 | Extracellular | Soluble | 0 | 0.0002 | 0.8952 | 0 | 0.0001 | 0.0074 | 0 | 0 | 0.0971 | 0 |
| XP_018245899.1 | Extracellular | Soluble | 0 | 0.0001 | 0.9922 | 0 | 0.0001 | 0.0024 | 0 | 0 | 0.0051 | 0 |
| XP_018258159.1 | Extracellular | Soluble | 0 | 0.0001 | 0.9841 | 0.0001 | 0.0001 | 0.0066 | 0 | 0 | 0.009 | 0 |
| XP_018241799.1 | Extracellular | Soluble | 0 | 0.0002 | 0.974 | 0 | 0 | 0.0058 | 0 | 0 | 0.02 | 0 |
| XP_018236411.1 | Extracellular | Soluble | 0 | 0.0004 | 0.9646 | 0.0005 | 0 | 0.0063 | 0 | 0 | 0.0282 | 0 |
| XP_018239494.1 | Extracellular | Soluble | 0 | 0 | 0.9909 | 0 | 0.0005 | 0.003 | 0 | 0 | 0.0055 | 0 |
| XP_018232260.1 | Extracellular | Soluble | 0.0002 | 0.0074 | 0.7937 | 0.057 | 0.0026 | 0.0299 | 0.0615 | 0 | 0.0461 | 0.0017 |
| XP_018255330.1 | Extracellular | Soluble | 0 | 0 | 0.989 | 0 | 0.0001 | 0.0048 | 0 | 0 | 0.0061 | 0 |
| XP_018252222.1 | Extracellular | Soluble | 0 | 0.0001 | 0.9863 | 0 | 0.0001 | 0.0015 | 0 | 0 | 0.012 | 0 |
| XP_018257851.1 | Extracellular | Soluble | 0 | 0.0001 | 0.8081 | 0.0001 | 0.0087 | 0.057 | 0 | 0.0039 | 0.1222 | 0 |
| XP_018254522.1 | Extracellular | Soluble | 0 | 0 | 0.9822 | 0 | 0.0007 | 0.0051 | 0 | 0 | 0.012 | 0 |
| XP_018255725.1 | Extracellular | Soluble | 0 | 0 | 0.9822 | 0 | 0.0007 | 0.0051 | 0 | 0 | 0.012 | 0 |
| XP_018251424.1 | Extracellular | Soluble | 0.0001 | 0.0006 | 0.928 | 0.0004 | 0.0343 | 0.0271 | 0 | 0.0003 | 0.0091 | 0 |
| XP_018257788.1 | Extracellular | Soluble | 0 | 0 | 0.8105 | 0 | 0.0027 | 0.1501 | 0 | 0.0013 | 0.0354 | 0 |
| XP_018254196.1 | Extracellular | Soluble | 0 | 0.0004 | 0.7045 | 0.0001 | 0.0038 | 0.087 | 0 | 0.0012 | 0.203 | 0 |
| XP_018258804.1 | Extracellular | Soluble | 0 | 0.0001 | 0.9897 | 0 | 0.0015 | 0.0057 | 0 | 0.0002 | 0.0029 | 0 |
| XP_018252399.1 | Lysosome/Vacuole | Soluble | 0 | 0.0062 | 0.4249 | 0.0002 | 0.0001 | 0.1202 | 0.0001 | 0 | 0.4482 | 0.0001 |

**Supplementary table S7:** Subcellular localization of the 56 differentially expressed CAZymes with their protein accessions coding for secretory peptides.

**Supplementary figures:**


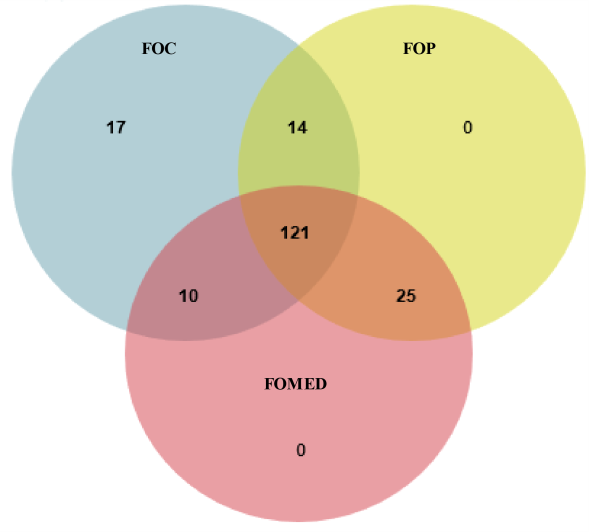


**Figure S1.** Orthologcluters of three legumes infecting ff. spp
(**FOMED**: *F.oxysporum* f. sp. *medicaginis*, **FOP**: *F.oxysporum* f. sp. *pisi*, **FOC**: *F.oxysporum* f. sp. *ciceris*)


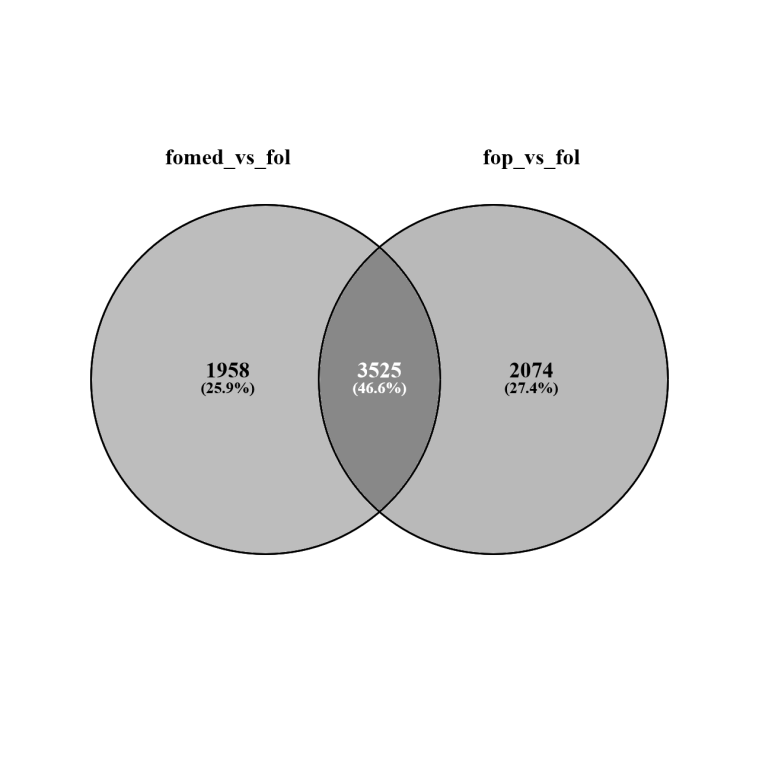


**Figure S2**: Comparision of differentially expressed genes
(**FOMED**: *F.oxysporum* f. sp. *medicaginis*, **FOP**: *F.oxysporum* f. sp. *pisi*, **FOC**: *F.oxysporum* f. sp. *ciceris*)
